# The dynamic dimer structure of the chaperone Trigger Factor

**DOI:** 10.1038/s41467-017-02196-7

**Published:** 2017-12-08

**Authors:** Leonor Morgado, Björn M. Burmann, Timothy Sharpe, Adam Mazur, Sebastian Hiller

**Affiliations:** 1 Biozentrum, University of Basel, Klingelbergstrasse 70, 4056 Basel, Switzerland; 2 Present Address: Department of Chemistry and Molecular Biology, Wallenberg Centre of Molecular and Translational Medicine, University of Gothenburg, 405 30 Göteborg, Sweden

## Abstract

The chaperone Trigger Factor (TF) from *Escherichia coli* forms a dimer at cellular concentrations. While the monomer structure of TF is well known, the spatial arrangement of this dimeric chaperone storage form has remained unclear. Here, we determine its structure by a combination of high-resolution NMR spectroscopy and biophysical methods. TF forms a symmetric head-to-tail dimer, where the ribosome binding domain is in contact with the substrate binding domain, while the peptidyl-prolyl isomerase domain contributes only slightly to the dimer affinity. The dimer structure is highly dynamic, with the two ribosome binding domains populating a conformational ensemble in the center. These dynamics result from intermolecular *in trans* interactions of the TF client-binding site with the ribosome binding domain, which is conformationally frustrated in the absence of the ribosome. The avidity in the dimer structure explains how the dimeric state of TF can be monomerized also by weakly interacting clients.

## Abstract (web summary)

The bacterial chaperone Trigger Factor (TF) is a dynamic protein and its dimer structure is unknown. Here the authors present a protocol combining NMR, computational and biophysical methods for the structural characterization of large dynamic protein complexes and show that TF forms a symmetric head-to-tail dimer.

## Introduction

**Fig. 1:**
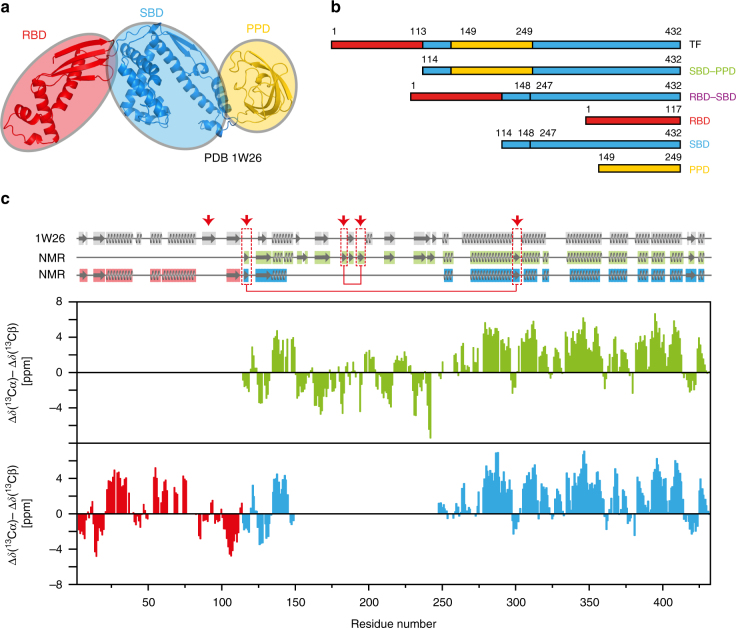
Domain organization of full-length TF and secondary structure elements in solution. **a** On the ribbon representation of a published TF crystal structure (PDB 1W26^8^), the three domains ribosome-binding domain (RBD), substrate-binding domain (SBD), and peptidyl-prolyl-*cis*/*trans* isomerase domain (PPD) are colored in red, blue, and yellow, respectively. **b** Domain constructs of *E. coli* TF used in this work. Six constructs of TF domains are shown with amino acid numbering corresponding to full-length TF. The names define a color code used throughout this work. **c** Secondary ^13^C chemical shifts plotted against the amino acid residue number of TF, as determined from triple-resonance experiments in the domain constructs SBD–PPD (green), RBD (red), and SBD (blue). A 1–2–1 weighting function for residues (*i*−1)–*i*–(*i* + 1) has been applied to the raw data to reduce noise and highlight regular secondary structure elements. Secondary structure elements were calculated for the crystal structure (PDB 1W26, gray) with DSSP^12^ and for the NMR data with CSI 3.0^13^ and are indicated on top. The red arrows and boxes highlight structural elements detected only in solution

The functionality of most proteins requires their correct folding subsequent to synthesis by the ribosome. Accumulation of structural intermediates or misfolded proteins into insoluble aggregates can lead to substantial impairment of cellular processes. Molecular chaperones have evolved in all kingdoms of life to play fundamental roles in protein biogenesis by preventing misfolding and aggregation of proteins, including transport of clients from their point of synthesis to their final cellular destination, where proper folding occurs^1–4^. Trigger Factor (TF) is a chaperone found in Gram-negative and Gram-positive bacteria as well as in chloroplasts^5^. TF binds to the translating ribosome and is thus the first chaperone to interact with newly synthesized polypeptides. TF is highly abundant in *Escherichia coli* cells but it is not essential for cell viability since its depletion is compensated by up-regulation of the functionally alternative chaperone DnaK^6^. However, the deletion of both chaperones is lethal at temperatures above 30 °C^6^. TF interacts with a multitude of substrates, among which outer membrane proteins are the most abundant ones, as revealed by ribosome profiling experiments^7^. TF consists of 432 amino acid residues and is organized in three domains adopting an overall elongated shape (Fig. 1a, b)^8^. The N-terminal domain (residues 1–113) is the ribosome-binding domain (RBD), that contains the TF signature motif GFRxGxxP (residues 43–50), via which TF binds to the ribosomal protein L23^9^. The peptidyl-prolyl isomerase domain (PPD) on the opposite side of TF can catalyze the isomerization of peptidyl-prolyl bonds and is structurally homologous to FK506-binding proteins^10^. The C-terminal domain of TF is the substrate-binding domain (SBD), stabilized by a linker between the RBD and PPD domains (residues 114–149)^11^. The SBD forms the central body of the protein and has two helical arms that create a cavity (Arm1: residues 302–360, Arm2: residues 361–412).

In the absence of ribosomes, TF is known to exist in a two-state equilibrium between a monomeric and a dimeric form. The dissociation constant (*K*_D_) of the dimer under physiological conditions is in the range 1–18 μM, as determined by multiple techniques^14, 15^. Since the cellular concentration of TF is 50 μM^5^, in the absence of excess concentrations of clients and ribosomes, the dimer is the dominant apo form of TF under physiological conditions, representing a pool of “resting state” molecules. However, despite numerous studies, the spatial arrangement of TF in its dimeric form has remained unclear. Several atomic resolution structures of TF from different organisms (*E. coli*, *Thermotoga maritima*, *Vibrio cholerae*, *Deinococcus radiodurans*, and *Mycoplasma genitalium*) are available for the full-length protein^8, 16, 17^, as well as for its individual domains^10, 11, 18, 19^, in complex with the ribosome^8, 20^ or in complex with substrates^17, 21^. While the fold of the individual domains is essentially maintained in these available crystal structures, their relative orientation varies substantially. Different dimeric arrangements are observed, some or most of which may have arisen from crystal contacts, and no consistently recurring dimeric arrangement is observed. For example, the TF *E. coli* crystal structure (PDB 1W26) shows a network of crystal contacts formed by the same residues that form the substrate-binding cradle but does not show an apparent dimer interface^8^. Or, the *T. maritima* structure was determined both in apo form (PDB 3GU0) and in complex with the ribosomal protein S7 (PDB 3GTY), resulting in different arrangements^17^. Furthermore, the structure of *V. cholerae* TF was determined on a construct with a C-terminal truncation that was later shown to diminish the dimerization and also the chaperone activity^22, 23^. Additionally, the dimerization interface was characterized by fluorescence resonance energy transfer and cross-linking experiments, and a nearly perpendicular orientation between the monomers was proposed^14, 24^. Notably, all observed TF dimer arrangements except in the *T. maritima* holo structure are asymmetric. An independent possibility for dimer formation could be extrapolated from the crystal structure of the isolated RBD (PDB 1OMS), which forms symmetric dimers^18, 25^, and modeling the full-length structure on this template would result in a symmetric arrangement. Finally, published small-angle X-ray scattering (SAXS) measurements show a compact globular particle with a length of about 37 Å^26^. Overall, different models and suggestions are thus available for the structure of the TF dimer.

This work aims to determine the structural arrangement of the *E. coli* TF dimer in aqueous solution at the atomic level. A combination of solution Nuclear Magnetic Resonance (NMR) experiments together with biophysical methods revealed that the arrangement of the TF dimer is caused by a dynamic interaction between two monomers. Specific NMR distance constraints established the structure of the TF dimer. Two conformers are presented and the experimental strategy is described, which will be generally useful to describe dynamic protein complexes.

## Results

### The domain folds are preserved in solution

To obtain a structural description of the TF dimer, we characterized full-length TF and individual domain constructs by high-resolution NMR spectroscopy. The individual domains, as well as the bi-domain construct SBD–PPD, gave rise to well-dispersed and high-quality spectra, enabling backbone resonance assignment in a sequence-specific manner using triple-resonance experiments together with information from published assignments (Supplementary Figs. 1 and 2)^21, 25^. Since the domain folds are preserved in all available TF crystal structures, we decided to probe the integrity of individual folds in aqueous solution by calculating the secondary chemical shifts of the RBD, SBD, and SBD–PPD constructs and comparing them to the available TF crystal structure (PDB 1W26; Fig. 1c). Compared to the crystal structure, most of the secondary structure elements are maintained in solution, with two notable differences. As previously shown by Hsu and Dobson^25^, the third β-strand of the RBD is not detected in solution. This strand is at the edge of the β-sheet element of the RBD and is possibly disordered in solution. On the other hand, two short β-strand pairs are formed by residues 115–117 with 298–301, and by residues 181–183 with 192–195, respectively. The former segments face each other in the linkers connecting the RBD to the SBD and the linker between the SBD body and one of the arms. The latter are located within the PPD. Overall, the data show that the structures of the individual domains are maintained in solution.

### The topology of the TF dimer

As a next step, we quantified the pairwise homotypic and heterotypic interactions of individual TF domain constructs. Size exclusion chromatography coupled with multi-angle light scattering (SEC–MALS) experiments were used to determine the homo-oligomerization of the constructs (Table 1 and Supplementary Fig. 3). The accessible range of protein concentrations was limited by the individual solubilities of the domain constructs and by the dilution factor of the SEC–MALS experiments. However, since MALS data for dimerization can be fitted with constrained values for the titration end point masses, *K*_D_ values or lower limits thereof can be reliably obtained also from solubility-limited data sets. The data reveal that two of the six tested constructs, full-length TF and RBD–SBD, undergo a monomer–dimer transition in the micro-molar concentration range in solution, while the other four domain constructs, RBD, SBD, PPD, and SBD–PPD, do not homo-oligomerize in the concentration range analyzed. For the two constructs that form a dimer, TF and RBD–SBD, the respective dissociation constants *K*_D_ were additionally measured by sedimentation equilibrium analytical centrifugation (AUC) and the values agree with those obtained by SEC–MALS. Under our experimental conditions, full-length TF formed a dimer with *K*_D_ = 2.5 ± 1.1 μM, and the RBD–SBD construct with *K*_D_ = 63 ± 13 μM, as averaged from the independent methods (Table 1). We also determined the impact of the buffer ionic strength on the dimerization (Supplementary Fig. 3 and Table 1). For both full-length TF and the RBD–SBD construct, the dimer affinity decreased with increasing potassium chloride concentration, indicating that the interaction includes substantial electrostatic components. Importantly, all these experiments were performed on protein constructs lacking any His_6_-purification tag. Preliminary SEC–MALS data collected for His_6_-tagged full-length TF showed that the presence of such tags artificially increases the dimerization affinity. In contrast to the wild-type, the His_6_-tagged construct was still completely dimeric at an elution concentration of 10 µM, which means, with a conservative estimate, its *K*_D_ must be below 100 nM, at least one order of magnitude lower than for untagged full-length TF (Supplementary Fig. 4).

**Table 1:** Dimer dissociation constants *K*_D_ (μM) of TF domain constructs

**Domain construct**	**AUC**	**SEC–MALS**		
	**100 mM KCl**	**100 mM KCl**	**250 mM KCl**	**500 mM KCl**
TF	3.3 ± 0.6	1.7 ± 0.2	9 ± 2	30 ± 8
RBD–SBD	54 ± 6	72 ± 16	>500	>500
SBD–PPD	n.d.	>1000	n.d.	n.d.
RBD	n.d.	≥1500	n.d.	n.d.
SBD	n.d.	>1000	n.d.	n.d.
PPD	n.d.	>5000	n.d.	n.d.

n.d., not determined

**Fig. 2:**
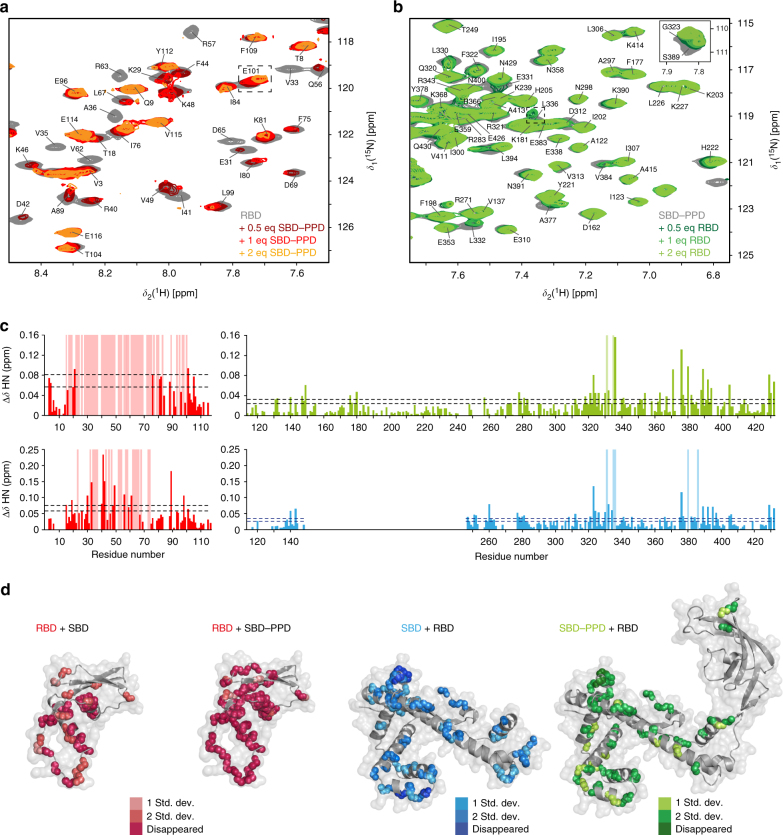
Localization of pairwise interaction sites on individual TF domains. **a** NMR titration of unlabeled SBD–PPD to 100 μM [*U*-^15^N] RBD in sample buffer (20 mM K-phosphate pH 6.5, 100 mM KCl, 0.5 mM EDTA) at 25 °C and 700 MHz. **b** NMR titration of unlabeled RBD to 250 μM [*U*-^2^H,^15^N] SBD–PPD in sample buffer at 25 °C and 700 MHz. **c** Chemical shift perturbation of the amide moieties observed in the titrations: [*U*-^15^N] RBD + SBD–PPD (top left), [*U*-^15^N] RBD + SBD (bottom left), [*U*-^2^H,^15^N] SBD–PPD + RBD (top right), and [*U*-^2^H,^15^N] SBD + RBD (bottom right). Light-shaded bars represent peaks undergoing line-broadening. Dashed lines are plotted at defined thresholds (mean value of the chemical shift perturbations plus one time and plus two times the standard deviation corrected to zero). **d** Significant chemical shift perturbations plotted in TF crystal structure (PDB 1W26). The affected residues are plotted with color gradient from light to dark for peaks with chemical shift changes above the threshold and that broaden beyond detection, respectively

The heterotypic domain interactions were probed by solution NMR chemical shift titrations, in which one isotope-labeled domain was titrated with the second unlabeled domain (Fig. 2, Supplementary Table 1, and Supplementary Fig. 5). Out of six possible pairwise combinations of the domain constructs RBD, SBD, PPD, and SBD–PPD, two gave rise to detectable interactions in the micro-molar concentration range. Overall, the biophysical characterization of the domains thus yielded an interaction matrix, representing the dimer topology of TF (Supplementary Table 1). These interactions of the TF domains are consistent with a single possible arrangement wherein the dimer of TF is formed by intermolecular contacts between the RBD of one protomer and the SBD of the other protomer, and that the resulting core interaction is mildly stabilized by the presence of the PPD.

### The RBD is dynamic in the TF dimer

The NMR spectra of full-length TF share the notable feature that the resonances of the RBD are mostly absent, i.e., the 2D [^15^N,^1^H]-TROSY spectrum of full-length TF is essentially a superposition of the two isolated domains SBD and PPD (Supplementary Fig. 6). In the spectrum, a single set of resonances was observed, indicating that the two protomers in the dimer are structurally equivalent at least for the SBD and PPD. A closer inspection of the full-length TF NMR spectrum, together with the sequence-specific resonance assignments, revealed that only 16 out of 108 expected resonances of the RBD are observed. Ten of these could be unambiguously assigned (residues 7, 10, 11, 12, 16, 17, 105, 107, 108, 111), and these are all located in the β-sheet region in the proximity of the SBD. Furthermore, some resonances of the SBD are line-broadened in full-length TF (Supplementary Fig. 6). A similar effect was observed for the construct RBD–SBD, which features an NMR spectrum highly similar to the spectrum of SBD (Supplementary Fig. 6). Thus, the NMR resonance lines of most of the RBD and some of the SBD are line-broadened as soon as the RBD is in contact with the SBD, both in the full-length TF and the RBD–SBD construct. The line-broadening directly indicates the presence of dynamic processes on the NMR intermediate timescale, i.e., with kinetic rates between 1000 and 10 s^−1^ ^27,28^.

**Fig. 3:**
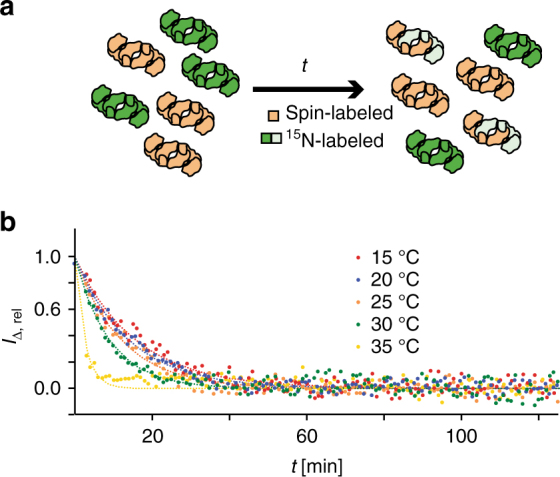
Determination of the lifetime of the TF dimer. **a** Experimental scheme. At the onset of the experiment (*t* = 0), separately produced samples of [*U*-^15^N,^2^H]-labeled (green) and randomly spin-labeled (brown) TF are mixed in equal ratio in sample buffer (left). The NMR signal intensity is then monitored in real time *t* during the equilibration to the end point (right). NMR signals of protomers bound to a spin-labeled protomer are reduced in intensity by the intermolecular PRE, symbolized by light green color. **b** Intensity of ^15^N-filtered NMR signals, *I*_Δ, rel_, following the experimental scheme in **a**. The lifetime of the TF dimer is obtained by non-linear least-square fits (lines) to the data (dots). See Supplementary Note 1 for mathematical details. The experiment was performed at five temperatures in the range 15–35 °C, as indicated

In the monomer–dimer equilibrium of TF, these intermediate timescale dynamics could a priori be caused by three different kinetic processes, or combinations thereof: (i) conformational exchange within the monomeric species, (ii) the transition of a protomer between its monomeric and its dimeric form, or (iii) conformational exchange within the dimeric species without going through a monomeric species. Since the individual domains of TF in their monomeric forms, as well as monomerized mutants of TF (see below), do not feature the observed line-broadening, the process (i) does not have strong contributions to the observed line-broadening in the TF dimer. We then used a spin-label exchange experiment in real time, to measure the exchange rate of the dimer to monomer equilibrium as a function of temperature, and thus the possible impact of process (ii). The data show that the lifetimes of the TF dimer is 15.8 min at 15 °C and 2.6 min at 35 °C, corresponding to dimer dissociation rate constants of *k*_off_ = 0.0011 s^−1^ and *k*_off_ = 0.006 s^−1^, respectively (Fig. 3). Lifetimes determined at further intermediate temperatures are within this range. These monomer–dimer exchange kinetics are not close to the intermediate exchange regime, thus leaving the process (iii) as a main mechanistic cause for the observed intermediate exchange of the TF dimer. Overall, the RBD thus populates a conformational ensemble in the TF dimer, with individual conformers connected by exchange rate constants on the intermediate timescale.

### Spatial domain positioning in the dimeric arrangement

To establish a structural model of the dynamic TF dimer, we obtained experimental constraints from two sources: chemical shift perturbation (CSP) experiments and paramagnetic relaxation enhancements (PREs). To this end, the contact interfaces of the interacting domains RBD and SBD were mapped by NMR CSP titrations (Fig. 2). Significant CSPs on the RBD were observed upon titrating with either SBD or with the bi-domain construct SBD–PPD. Significantly shifting resonances are, for example, residues E101, L336, and S389 (Fig. 2). Thereby, multiple peaks of the RBD disappeared beyond detection, with the effect being more pronounced in the titration with SBD–PPD. This result agrees with the spectroscopic observations on full-length TF, where only few peaks for the RBD are observable. On the SBD, large chemical shift changes were observed upon titrating RBD to either SBD alone or SBD–PPD, with the strongest changes located in the tips of the arms of the SBD (residues 323/331/335/336 and 376/380/386). The resonances of some residues in the SBD also broadened beyond detection, revealing the changes in local protein dynamics upon interaction. No CSP effect was observed in any of the titrations with the PPD only (Supplementary Table 1). These titration experiments establish the location of the main interaction surfaces in the individual domains for molecular docking.

**Fig. 4:**
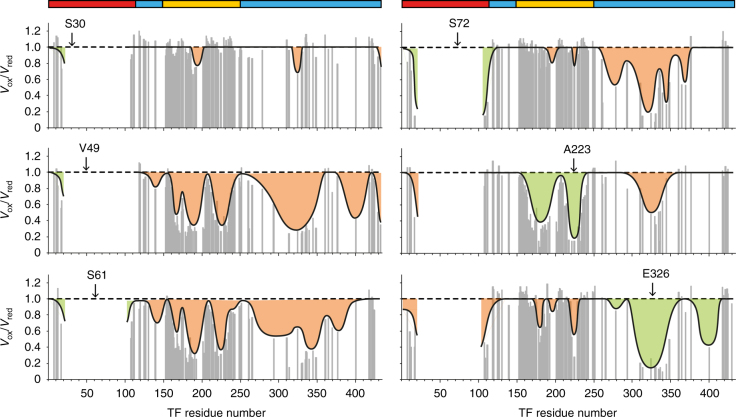
Domain contacts in the full-length TF dimer. Result of PRE experiments with a paramagnetic spin label attached to one of the positions S30, V49, S61, S72, A223, and E326 in full-length TF measured in sample buffer (20 mM K-phosphate pH 6.5, 100 mM KCl, 0.5 mM EDTA) at 25 °C and 700 MHz. The peak volume ratio between oxidized and reduced samples from 2D [^15^N,^1^H]-TROSY is plotted against the residue number. For visualization purposes, a value of 0.15 is shown for the peaks that were broadened beyond detection in the paramagnetic sample. Data are shown only for non-overlapping resonances. The black line outlines the PRE effect observed for each mutant. The orange-shaded regions correspond to intermolecular PRE and the green shaded to intramolecular effects, as expected from the monomeric crystal structure (PDB 1W26). The colored bars on top show the sequence domain organization as in Fig. 1

To obtain atomic distance information on such a dynamic interaction with line-broadened peaks, the method of choice is the use of PREs. For these measurements, a spin label with a paramagnetic unpaired electron is introduced at selected sites into full-length TF, causing enhanced relaxation of the nuclear spins in its vicinity up to ∼25 Å, and thus providing long-distance information (Fig. 4). In general, one possibility to detect purely intermolecular PRE distance information is the use of mixed samples, such as a 1:1 mixture of isotope-labeled (e.g., ^15^N) and spin-labeled protein (SL). However, the spectral analysis with these type of preparations is not straightforward because three types of dimers are present in solution (^15^N/^15^N, ^15^N/SL, and SL/SL) with overall decreased experimental sensitivity and a reduced averaged effect^29^. Our initial tests showed that the complicated NMR spectrum, the large molecular weight of TF with 432 residues, as well as its limited solubility make this approach not feasible for TF. Therefore, we decided to work with a sample containing a single species of uniformly isotope- and spin-labeled TF.

The positioning of the spin label was chosen as to maximize the available structural information. PREs were measured in TF samples with the spin label in the following positions: S30C, V49C, S61C, and S72C in the RBD, A223C in the PPD, and E326C in the SBD. Importantly, placing multiple probes at the line-broadened RBD was essential to obtain distance information between this domain and the other residues in the molecule, as the reverse experiment with detection on the RBD would not be meaningful. The PRE data were quantitatively analyzed for the residues in full-length TF with unambiguous assignment and non-overlapping peaks (Fig. 4). For consecutive polypeptide segments, the observed PRE effects were then classified by comparing the measured interspin distances with the known domain structures. Those PREs that could be explained based on the monomeric structure were classified as intramolecular (highlighted in green on Fig. 4) and all remaining PREs as intermolecular effects (highlighted in orange on Fig. 4). Among the multiple long-range distance contacts observed, the most striking one was verified between the spin-labeled residues V49C or S61C in the RBD and the inner surface of the PPD (regions around residues 165, 190, and 220). These regions are far apart in the TF monomer structure but close in the dimer, independently confirming the previous conclusion that the PPD of one protomer is in close spatial contact with the RBD of the other protomer.

### Structure of the TF dimer

**Fig. 5:**
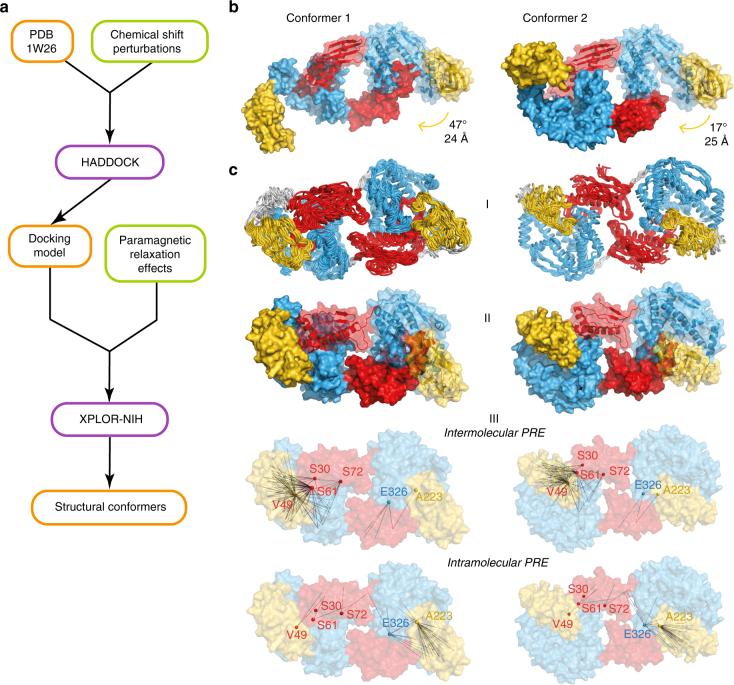
Structure of the TF dimer in solution. **a** Flowchart for structural model determination of TF dimer. Structural models are indicated in orange boxes. Experimental data contributions are indicated in green boxes. Software packages are identified in purple boxes. **b** Lowest energy structures from the two clusters obtained with HADDOCK docking based on chemical shift perturbation data. Both monomers are represented in surface view, and one of them is semi-transparent to show the backbone. **c** XPLOR-NIH results represented as (**I**) ensemble of the 10 lowest energy structures with the flexible residue segments in gray, (**II**) lowest energy structures with both monomers represented in surface view, one of them depicted semi-transparent to show the backbone, and (**III**) lowest energy structures in surface representation with experimental PRE distances represented, intermolecular (A–B, top) and intramolecular (A–A, bottom)

The experimental data from the CSP and PRE measurements were then used to calculate structural conformers of the TF dimer. Based on the domain folds of the crystal structure 1W26, which we had validated by secondary chemical shift analysis, we employed a two-step procedure consisting of a CSP-based docking followed by PRE-driven annealing (Fig. 5a). For the first calculation step, the docking algorithm HADDOCK^30–32^ was employed, using the CSP data between the RBD and the SBD as input, together with C_2_ symmetry restraints for the two protomers (Supplementary Table 2). The output of the HADDOCK calculation contained two structural clusters with similar target energy function and overall identical domain topology (Fig. 5b). In conformer 1, the RBD locates inside the cavity formed by the SBD arms. In this structure, the ribosome-binding site is completely occluded inside the SBD cavity. In conformer 2, the RBD of one protomer lies on top of the tips of the arms of the SBD of the other protomer, with the ribosome-binding motif in close contact with one of the arms.

In the second calculation step, the results from HADDOCK together with the PRE data were used as input for the structure calculation software XPLOR-NIH^33^ (Supplementary Table 3). Based on the experimental confirmation that the individual domains feature the secondary structure elements known from the crystal structure, a set of distance restraints were introduced to maintain the geometry of these elements, referred to as elastic fold network (EFN) constraints. The medium-range PRE restraints were used as intra- or intermolecular, and the long-range PRE restraints were used as intra- and intermolecular. Thereby, approximately 75% of the 521 PRE restraints were intermolecular, providing sufficient information for the calculation of the arrangement of the protomers. Both calculated structural conformers share the same overall topology and each fits the experimental data similarly (Fig. 5c and Supplementary Table 4). In both models, the SBD preserves the relative position of its architectural elements, thus maintaining the central cavity. Comparing with the crystal structure 1W26, the PPD rotates toward its own SBD by 47° and 24 Å in model 1, and by 17° and 25 Å in model 2, getting close to the RBD of the other monomer. In model 2, the RBD sitting on top of the SBD arms after the docking step has the loop that contains the ribosome-binding site (residues 42–49) located between the SBD arms after the XPLOR annealing. Notably, the conformers each fulfill a large majority, but not exactly all experimental constraints (Supplementary Table 4). We thus propose that these two structures describe two representative conformers in the conformational equilibrium of dimeric TF.

### Experimental validation of the models

To validate the structures and their interaction mode, we created variants of TF with amino acid residue mutations at selected positions in the dimer domain interfaces. On the basis of a TF variant with reduced ribosome binding^9^, we introduced three sets of mutations, either isolated or in combination. The mutation sets mutB and mutC, previously described by Saio et al.^21^, are located in one arm and the neck region of TF, respectively, while a newly designed set of mutations, mutD, was chosen to be located at the other arm (Supplementary Fig. 7a). The mutant mutB has four hydrophobic amino acids substituted by alanines in Arm2 and mutC has a single hydrophobic amino acid substituted in the SBD cavity. These mutants were previously shown to have reduced chaperone activity in the form of lower anti-aggregation activity^21^. The mutant mutD has two charged residues mutated to alanines in Arm1. All mutants were expressed, purified, and their monomer–dimer equilibrium analyzed by SEC–MALS (Supplementary Fig. 7 and Supplementary Table 5). All three mutant sets weakened the dimer affinity, with mutC and mutD having a moderate effect, while mutB completely abrogated the dimerization, resulting in monomeric protein even at the highest examined concentration. As expected from their distant location in the structure, the mutation sets mutC and mutD showed additive effects in the dimerization as their joint incorporation lead to a further weakened affinity (Supplementary Table 5). Finally, an inspection of the 2D [^15^N,^1^H]-TROSY NMR spectra of the mutant TF proteins showed that the monomerization directly lead to the appearance of the resonance peaks of the RBD (Supplementary Fig. 8), in full agreement with the finding that the line-broadening of the RBD is caused by the exchange dynamics of the RBD inside the SBD cavity. In the spectra of mutB and mutB + mutC, all resonances of the RBD, except G95 and A27, could be identified; the latter resonances are however already quite weak in isolated RBD. This appearance of RDB signals is highlighted for residue Ile19 (Supplementary Fig. 8). Overall, on the one hand, the structural location of the mutation sets validates the contact sites of the dimer and thus our structural conformers. On the other hand, the observation that the same mutations which are known to decrease the chaperoning activity of TF also lead to monomerization, confirms that the dimerization between two TF molecules arises from client-like *in trans* self-interactions.

## Discussion

**Fig. 6:**
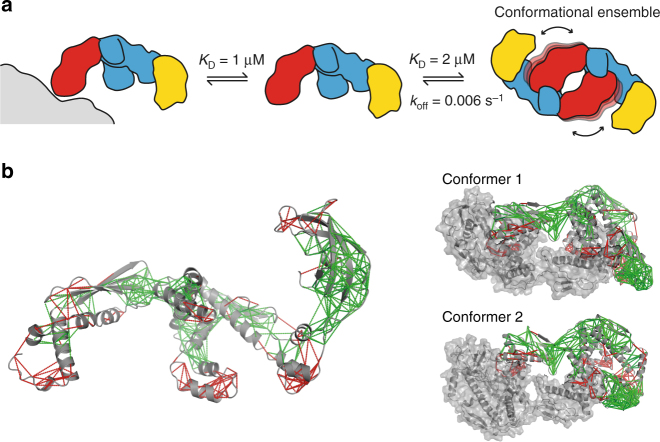
Equilibrium and frustration of the TF dimer in solution. **a** TF dimer is highly dynamic and is in equilibrium in solution with its monomeric form and with its ribosome-bound form. The ribosome is represented in gray. **b** Frustration analysis of TF. Local frustration for TF crystal structure was calculated with the online tool Protein Frustratometer 2^34^ (PDB 1W26) and is plotted on TF crystal structure (left) and on the dimer structural models (right). Minimally frustrated interactions are depicted as green lines, highly frustrated interactions as red lines

The experiments presented in this work have resolved the long-standing question about the spatial arrangement of the dimeric form of *E. coli* TF in solution. The dimer arrangement is dynamic, with the two RBD domains populating a conformational ensemble in the center of the complex. The arrangement results from intermolecular *in trans* interactions of the TF client-binding site with the RBD. In the absence of clients, TF is in three-state equilibrium between a ribosome-bound, a monomeric, and a dimeric form (Fig. 6). The structure of the dimer is a dynamic conformational equilibrium. Importantly, the dimeric structure provides an explanation why the TF dimer, which has an apparent dimeric affinity of 2.5 μM, can be monomerized by clients with weaker affinities, such as different PhoA-derived model substrates with affinities in the range ∼2–14 μM^21^. Since the dimerization affinity results from two weaker interactions between the SBDs of each protomer and the RBDs of the respective other by avidity, monomerization can be readily achieved by a client with weaker global, but higher local affinity. Clients with weaker affinity than the local interaction will however not bind. The dimeric state thus also provides a selectivity filter for very weakly interacting clients, protecting the TF client sites from promiscuous binding.

Our methodological approach to describe the structure of the TF dimer is adapted to the dynamicity of the complex, where the RBD adopts a dynamic ensemble state, rather than a single conformation. Intermolecular nuclear Overhauser effects (NOEs) of dynamic ensembles can be difficult or impossible to interpret^35^, and in such situations, PREs are thus the method of choice to obtain intermolecular spatial correlations. Importantly, despite the unobservability of the RBD by NMR, positioning paramagnetic probes in this domain allowed measuring intermolecular distances between this otherwise invisible domain and the others. Together, the CSP and PRE data then allowed the determination of two structural models of the TF dimer in solution using the software packages HADDOCK and XPLOR-NIH. Refinement of the docking models with restraints that maintain the fold of each domain led to two models that have overall similarity and that each fulfill a large majority, but not exactly all experimental constraints. The two conformers are thus a first-order approximation to describe the conformational ensemble of TF dimer. With further experimental data that may potentially be available from additional experiments, it may become interesting to calculate a refined ensemble of the TF dimer with more representative conformers in the future.

When comparing the dimer structure to the available crystal structures, a similarity to the arrangement of the holo structure of *T. maritima* TF is directly observed^17^. In that structure, two molecules of TF associate and bind two natively folded molecules of the substrate S7, one residing inside each SBD cavity. It was also observed that in both apo structures from *T. maritima* and *E. coli*, the RBD of a symmetry-related molecule is bound within the SBD cavity. The observed flexibility, together with the dynamic behavior at the interface, may provide a rationale for why it has so far not been possible to crystallize the dimeric form in the biologically relevant conformation. Furthermore, the high flexibility of the linker between SBD and PPD observed by the conformers is in full agreement with a recent cryo-EM study of TF bound to ribosomes and nascent chains, where the position of the PPD had to be adjusted by 24° rotation toward the SBD to fit on the density map^20^, as well as with recent molecular dynamics studies showing that the domains maintain their secondary structure during simulations, but that the linkers between the domains are quite flexible^36–38^.

A recent study on the mechanism of recognition between multiple chaperones and the client protein Im7 indicated that chaperones identify locally frustrated regions on client proteins^39^. We analyzed the frustration of the TF crystal structure^34^ to rationalize why TF forms dimers, and why the RBD binds in the substrate binding cavity (Fig. 6b). Several distinct regions of the protein are shown to be highly frustrated, namely the RBD loop containing the signature motif, the tips of the SBD arms, and the linker between the SBD and the PPD. Intriguingly, in the structural models of dimer, these regions all interact with each other, suggesting that the release of frustration energy is a driving force behind the TF dimer formation (Fig. 6b). This force contains both hydrophobic and electrostatic components, in agreement with our salt-concentration-dependent SEC–MALS experiments. TF recognizes the frustrated RBD in the absence of the ribosome as if it was a client protein. The *in trans* self-interaction of TF thus follows general laws of chaperone–client interactions.

## Methods

### Protein preparation

TF (full length, residues 1–432) was cloned from *E. coli* genomic DNA with NdeI and NotI restriction sites and ligated into a pET28b expression vector containing a thrombin-cleavable N-terminal His tag. All primer sequences used in this work are shown in Supplementary Table 6. RBD (residues 1–117) was constructed by introducing a stop codon at position 118 by site-directed mutagenesis. The remaining constructs, SBD (114–148/247–432), PPD (149–249), RBD–SBD (1–148/247–432), and SBD–PPD (114–432), were prepared by restriction-free cloning^40^. The thrombin cleavage site was mutated for a TEV cleavage site by site-directed mutagenesis. BL21 (λ DE3) pLysS (Novagen) cells were transformed with the plasmids and grown at 37 °C in medium containing 30 μg ml^−1^ kanamycin to OD_600_ = 0.5–0.6 and then for 30 min at 25 °C. Expression was induced by 0.4 mM IPTG (Apollo Scientific) and cells were harvested 15–18 h after induction by centrifugation for 15 min at 6000×*g*. For RBD, BL21 (λ DE3) Lemo (Novagen) cells were used, and the protein was expressed as inclusion bodies. Cells were resuspended in purification buffer (25 mM HEPES pH 7.5, 150 mM NaCl) supplemented with 10 mM imidazole and Complete EDTA-free protease inhibitor (Roche), lysed by French press, and centrifuged for 45 min at 38,000×*g* at 4 °C. The supernatant was applied to a Ni-HisTrap column (GE Healthcare) and eluted with an imidazole gradient in purification buffer with 500 mM NaCl. The proteins eluted at 80–150 mM imidazole. The fractions containing the protein were dialyzed against purification buffer and subsequently denatured by the addition of 6 M guanidine hydrochloride (Gdm-HCl). For the RBD, the pellet containing the inclusion bodies after cells lysis was resuspended in purification buffer supplemented with 6 M Gdm-HCl. The denatured proteins were applied to Ni^2+^-beads (Genscript) and incubated for 1 h under continuous shaking. The resin was extensively washed with purification buffer with 6 M Gdm-HCl and the proteins eluted with the same buffer containing 200 mM imidazole. Eluted proteins were refolded by dialysis against 50 mM TRIS pH 8. Then, 1 mM DTT and 0.5 mM EDTA were added to the samples for TEV-protease cleavage overnight at 4 °C (ratio TEV:protein 1:30 mg). After cleavage, proteins were dialyzed against purification buffer, denatured by the addition of 6 M Gdm-HCl, and further applied to Ni-charged beads to separate from the TEV protease and the cleaved tag. After incubation for 1 h, the flow-through and wash containing the cleaved protein were refolded by dialysis in purification buffer. Proteins were concentrated by ultracentrifugation (Vivaspin concentrators, Sartorius) before being applied to a gel filtration column (HiLoad 16/600 Superdex 200 pg or Superdex 75 pg, GE Healthcare) equilibrated with 20 mM K-phosphate pH 6.5, 100 mM KCl, 0.5 mM EDTA (sample buffer). For NMR experiments, the proteins were expressed in M9 minimal media containing the desired isotopes (H_2_O or D_2_O supplemented with ^15^NH_4_Cl and D-glucose for double labeling or (^2^H,^13^C)-D-glucose for triple labeling).

### Random spin labeling of lysine residues

For spin labeling of the ε-amino groups with OXYL-1-NHS (1-oxyl-2,2,5,5-tetramethylpyrroline-3-carboxylate-N-hydroximide ester; Toronto Research Chemicals), TF was exchanged to spin-labeling buffer (10 mM sodium carbonate, pH 9.3) by using a PD-10 desalting column (GE Healthcare) according to the manufacturer’s instructions. A 10-fold molar excess of OXYL-1-NHS dissolved in DMSO was directly added to the protein solution, followed by incubation in the dark for 1 h at room temperature and additionally 2 h at 4 °C. To remove excess spin label and to exchange the samples into sample buffer, samples were washed with 20 volumes of sample buffer using Vivaspin concentrators (Sartorius, MWCO 30 kDa).

### Preparation of TF mutants

The single-point mutations S30C, V49C, S61C, S72C, A223C, and E326C for PRE measurements were introduced in the TF wild-type sequence by site-directed mutagenesis. The expression and purification of the mutant cysteine-containing proteins was performed as described for the wild-type protein, except that 1 mM DTT was added for the refolding and to the sample buffer for gel filtration. For validation of the structural models, the TF(3A) variant TF(F44A, R45A, K46A), which is deficient of the ribosome interaction^9^, was chosen as background. On this basis, three sets of mutations—mutB, mutC, mutD—were introduced, either as single sets or in combination. The mutation sets mutB (M374A, Y378A, V384A, F387A) and mutC (M140E) had previously been designed and characterized^21^, and the mutation set mutD (R316A, R321A) was newly designed.

### Spin labeling of cysteine mutants

The cysteine mutants of TF were spin labeled with MTSL ((1-oxyl-2,2,5,5-tetramethyl-Δ3-pyrroline-3-methyl)-methanethiosulfonate; Toronto Research Chemicals). Protein solution in sample buffer with DTT was exchanged to sample buffer with 500 mM KCl and without DTT using PD-10 desalting columns (GE Healthcare). A 10-fold molar excess of MTSL dissolved in acetonitrile was added to the protein and incubated for 1 h on ice and then overnight at room temperature, always in the dark. To remove unreacted MTSL, the buffer was exchanged to sample buffer. Sodium ascorbate was used for the reduction of the spin label in the NMR tube to a final concentration of 5 mM from a 500 mM stock solution.

### SEC–MALS

For SEC–MALS measurements, samples were separated at 6 °C for the individual domains, and at 26 °C for the full-length mutants, in sample buffer using a GE Healthcare Superdex 200 5/150 GL column or a Wyatt silica SEC column (4.6 × 300 mm, 5 μm bead, 150 Å pore) run on an Agilent 1260 HPLC. Elution was monitored by three detectors in series: (1) an Agilent multi-wavelength absorbance detector (absorbance at 280 and 254 nm), (2) a Wyatt Heleos II 8+ multi-angle light scattering (MALS) detector, and (3) a Wyatt Optilab rEX differential refractive index (dRI) detector. The columns were equilibrated overnight in the running buffer to obtain stable baseline signals from the detectors before data collection. Molar mass, elution concentration, and mass distributions of the samples were calculated using the ASTRA 6 software (Wyatt Technology). Inter-detector delay volumes, band broadening corrections, and light-scattering detector normalization were calibrated regularly using a 2 mg ml^−1^ BSA solution (ThermoPierce) and standard protocols in ASTRA 6.

For all constructs, essentially identical mass values were calculated using either dRI or absorbance signals to determine protein concentration, at concentrations where the comparison could be made. However, for the full-length TF, full-length mutants, RBD–SBD, SBD–PPD, and SBD concentration series, absorbance data were used in preference for molar mass calculations due to greater signal-to-noise at low concentration. In these cases, band-broadening corrections for the absorbance signal were obtained from the BSA monomer peak using the MALS detector as the reference. For the RBD and PPD series that used higher loading concentrations, dRI data were used to measure concentration as the absorbance signal was outside the linear range of the detector. In these cases, band-broadening corrections for the UV and MALS detector signals were obtained from the BSA monomer peak using the dRI detector as the reference.

For all constructs, the elution profiles exhibited a single major peak. For full-length TF; the full-length mutants 3A, mutC, and mutD; RBD–SBD and RBD sample series, the elution volume of the peak and the SEC–MALS-derived weight-averaged molar mass (*M*_w_) changed in a concentration-dependent manner. In all cases, *M*_w_ varied within the limits of the molar mass expected for the monomer and dimer, consistent with the presence of a fast-exchanging monomer–dimer equilibrium. In these cases, values of *M*_w_ and elution concentration at the top of the elution peak were used to fit the dissociation constant (*K*_D_) for each construct, assuming a fast monomer–dimer equilibrium (Eq. 1).

1 $$M_{\rm W} = 2M - M\frac{{ - K_{\rm D} + \sqrt {K_{\rm D}^2 + 8\left[ M \right]K_{\rm D}} }}{{4\left[ M \right]}},$$

where *M* is the molar mass of the monomer and [*M*] the molar concentration of the sample (in terms of monomer) as it passes through the MALS detector after elution from the column^41^.

For the series where concentration was measured by absorbance, the concentration at the MALS detector could be obtained directly from the absorbance signal after band-broadening correction (with the MALS detector as the reference instrument). However, for the RBD concentration series, the concentration measured at the dRI detector was lower than that at the MALS detector, due to significant band-broadening at the dRI detector. To obtain the correct concentration at the MALS detector for fitting the RBD data to Eq. (1), the elution concentrations calculated from the dRI data were multiplied by an experimentally determined factor of 1.26. 95% confidence intervals were determined from the fitting error in GraphPad Prism.

### Analytical ultracentrifugation

Sedimentation equilibrium runs were conducted at 6 °C using An-60Ti rotor in a Beckman Coulter XL-I analytical ultracentrifuge, for full-length TF and RBD–SBD, which exhibited dimer formation in SEC–MALS experiments. Samples were prepared at 12 μM for TF as well as at 48 and 135 μM for RBD–SBD, and the sample volume was 170 µl. Data were acquired at three speeds, 10,000 rpm (7800* g*), 16,000 rpm (20,000* g*), and 20,000 rpm (31,000* g*), with detection by radial absorbance scanning at 250 and 280 nm. At each, speed centrifugation was allowed to proceed until sedimentation equilibrium was attained, as judged by pairwise comparison of scans taken at 6 h intervals, using the approach to equilibrium function in the program Sedfit^42^. Equilibrium absorbance scans at three speeds (for both constructs) and two concentrations (for RBD–SBD) were globally fitted to a monomer–dimer model to obtain the dimer dissociation constant *K*_D_ using the program Sedphat^43^. Monomer masses and molar extinction coefficients at 280 nm for each construct were constrained to values calculated from their amino acid sequence, and the molar extinction coefficient for RBD–SBD at 250 nm was calculated from an absorbance spectrum and the extinction coefficient at 280 nm. Buffer density was calculated using Sednterp. The total concentration, bottom positions, RI noise, and baseline were globally fitted for each sample at multiple speeds, with mass conservation constraints employed. The globally fitted concentrations were in good agreement with the loading concentrations. 95% confidence intervals were determined using the automatic confidence interval search with projection method.

### NMR spectroscopy

All NMR samples were prepared in sample buffer. NMR experiments were recorded at 25 °C on Bruker Ascend 700 MHz and Bruker Avance III 900 MHz spectrometers, equipped with cryogenic triple-resonance probes. NMR data were processed with PROSA^44^ and analyzed with CARA and XEASY^45^. For sequence-specific backbone resonance assignment 2D [^15^N,^1^H]-TROSY^46^ and 3D TROSY-HNCACB^47^ experiments were acquired for [*U*-^2^H,^15^N,^13^C] TF, [*U*-^2^H,^15^N,^13^C] SBD–PPD, [*U*-^2^H,^15^N,^13^C] SBD, and [*U*-^2^H,^15^N,^13^C] RBD. For [*U*-^2^H,^15^N,^13^C] SBD–PPD, 3D TROSY-HNCA was also acquired, and for [*U*-^2^H,^15^N,^13^C] SBD, 3D TROSY-HNCA, 3D TROSY-HNCO, and 3D TROSY-HNCACO^47^. Assignment of [*U*-^15^N] PPD was obtained from the experiments for [*U*-^2^H,^15^N,^13^C] SBD–PPD. Secondary chemical shifts were calculated relative to the random coil values^48^. Titrations followed by 2D [^15^N,^1^H]-TROSY were performed between the monomeric constructs RBD, SBD, PPD, and SBD–PPD. The initial concentrations were 250 μM for SBD–PPD and 100 μM for the other constructs. The chemical shift changes of the amide resonances in the 2D [^15^N,^1^H]-TROSY spectra were calculated according to Eq. 2:

2 $${\mathrm{\Delta }}\delta \,{\rm HN} = \sqrt {\left( {{\mathrm{\Delta }}\delta \left( {\,^1{\rm H}}\right)} \right)^2 + \left( {0.2 \cdot {\mathrm{\Delta }}\delta \left( {\,^{15}{\rm N}} \right)} \right)^2}.$$

PRE experiments were performed on [*U*-^2^H,^15^N] TF at 300 μM. A 2D [^15^N,^1^H]-TROSY spectrum was first measured in the paramagnetic state, and after addition of ascorbate to the sample, the diamagnetic reference was measured (Supplementary Fig. 9). The volume of well-resolved peaks was measured with NEASY and used to calculate PRE rates (Eq. 3)^49^ that were further converted into distances (Eq. 4)^50^:

3 $$\frac{{V_{\rm ox}}}{{V_{\rm red}}} = e^{( - {\rm PRE}{\cdot}2\cdot\tau _{{\rm INEPT}})},$$

4 $$r = \root {6} \of {{\frac{K}{{{\rm PRE}}}\left( {4\tau _{\rm c} + \frac{{3\tau _{\rm c}}}{{1 + \omega _{\rm h}^2\tau _{\rm c}^2}}} \right)}},$$

where *r* is the distance between the electron and nuclear spins, *τ*_c_ is the correlation time for the electron–nuclear interaction (the approximation was made that *τ*_c_ is equal to the global correlation time of the protein determined by [^15^N,^1^H]-TRACT^51^, 42 ns), *ω*_h_ is the Larmor frequency of the nuclear spin (proton), and *K* is 1.23 · 10^−32^ cm^6^ s^−2^ ^50^. The secondary structure elements in solution were determined with the CSI 3.0 web server using Cα and Cβ chemical shifts^13^.

### Measuring of the lifetime of the TF dimer

About 100 μl of 100 µM [*U*-^2^H,^15^N] TF were mixed at *t* = 0 with 20 μl of D_2_O and 100 μl OXYL-1-NHS-labeled TF (100 μM). In real time after the mixing, single δ_1_[^15^N]-1D cross sections of 2D [^15^N,^1^H]-TROSY spectra with an experimental time of 60 s were acquired. The measurements were performed in 3 mm NMR tubes on an 800 MHz Bruker AVANCE III HD spectrometer equipped with 3 mm CP-TCI probe. The values for *t*_0_ (experimental dead time between mixing and acquisition of first data point) were 140 s (20 °C), 150 s (25 °C, 30 °C), and 160 s (15 °C, 35 °C), respectively, for the temperature-dependent measurements. For analysis of the data, the 1D proton signal intensity was integrated over the region 7.0–9.5 ppm using Topspin 3.5 (Bruker BioSpin). The data were fitted by least-square minimization to the equation $I_\Delta \left( t \right) = I\left( t \right) - I_\infty = \left( {I_0 - I_\infty } \right) \cdot \exp \left( { - t/\tau } \right)$, where *I*_0_ and *I*_∞_ are the NMR signal intensities at *t* = 0 and *t* = ∞, respectively, and *τ* is the global lifetime of the dimer (*τ* = *k*_off_^−1^). See Supplementary Note 1 and Supplementary Figure 10 for a derivation of this equation. Reference experiments, in which non-spin-labeled TF (instead of spin-labeled TF) was added to [*U*-^2^H,^15^N] TF, showed a constant signal intensity over the entire timescale, validating that the observed signal loss in the experiment with the spin label is due to the intermolecular PRE in the mixed dimer, caused by disassembly of the [*U*-^2^H,^15^N] TF dimer and the reassembly of the mixed dimer.

### Structure determination

The calculation of the structural model was performed in two phases: docking of the two monomers of TF using the CSP data and rearrangement of the individual domains using PRE distance restraints. In the first phase, the HADDOCK web server was used (Supplementary Table 2)^30–32^. The chain A of the *E. coli* crystal structure (PDB 1W26) was used for both monomers^8^. The active residues were defined as the residues having resonances that disappear or that had chemical shift changes above the mean plus one standard deviation corrected to zero^52^ in the titrations between RBD and SBD (Fig. 3). Passive residues were automatically defined by HADDOCK. Non-crystallographic symmetry (NCS) restraints and C_2_ symmetry restraints were imposed for all residues. Standard docking parameters were used. HADDOCK clustered 162 out of 200 calculated structures into 12 clusters (Supplementary Table 2). The top 2 HADDOCK models were used as input for XPLOR-NIH^33^ in the second phase.

EFN restraints were created with crystallography and NMR system (CNS) for each monomer in both HADDOCK models, following the selection rules of the DEN (deformable elastic network) method^53, 54^. EFN restraints differ from DEN in that they are not re-adjusted in the course of the structure calculation trajectory. The EFN restraints maintain the domains folded by constraining distances between 3 to 15 Å between atoms with a maximum sequence separation of 10 residues, and leave the linkers between the domains flexible. Based on published molecular dynamics simulations, the flexible linkers were defined for the residues 112–115, 149–155, and 241–261^38^. PRE restraints were introduced as distances between the Cβ atom of the mutated residue and the amide proton detected in the spectra. For each spectrum, an error was determined from the noise (as the standard deviation of the integrals of 12 peaks in the noise), and error propagation was applied to calculate errors for the final distances. The PRE restraints were divided into three classes, according to the ratio between the volume of the peaks in the paramagnetic and diamagnetic samples. For resonances with a volume ratio between 0.15 and 0.85, the calculated distance was restrained with ±4 Å margins. Only distances for which the propagated error was less than 1 Å were considered. For resonances with a volume ratio <0.15 or for which the resonances are broadened beyond detection, only an upper limit was defined. This limit was calculated for a ratio of 0.15 (16.12 Å) and given an upper limit of 4 Å. This selection resulted in 171 PRE distance restraints (Supplementary Table 2). Since these restraints can be intra- or intermolecular, these were submitted to XPLOR-NIH as ambiguous restraints. Resonances with ratio >0.85 were restrained only with a lower limit as the distance corresponding to ratio 0.85 (24.28 Å) with a lower limit of 4 Å. As no effect was observed for these resonances, these were restrained both as intra- and intermolecular (long-range restraints). The ±4 Å in distance constraints were previously shown to be sufficient to account for the flexibility of the MTSL tag and possible errors from the use of a global correlation time and the approximation of the intrinsic relaxation rates^50^. The structure calculation protocol was derived from the XPLOR-NIH gb1/anneal.py template script. The simulation was repeated 100 times and the 10 lowest energy structures were further refined in explicit solvent using gb1/wrefine.py script (Supplementary Table 3). The calculations were performed in torsion angle space except for initial and final minimization. The potential energy of the TF dimer was modeled by standard XPLOR-NIH bonded (bond, angle, dihedral and improper terms) and non-bonded (van der Waals term) potentials. The symmetry of the dimer was imposed by using distance symmetry and NCS restrains. The local geometry of TF domains was maintained by the EFN distance restraints. Water refinement was done using OPLSX parameters and the XPLOR-NIH Ramachandran potential (backbone dihedral angle database).

### Data availability

The two TF dimer conformers have been deposited in the PDB as entries 5OWI and 5OWJ and the NMR resonance assignments to the BMRB with accession codes 27239 and 27242. All other relevant data are available from the corresponding author upon reasonable request.

## Electronic supplementary material

Supplementary Information

## References

[1] Kim YE, Hipp MS, Bracher A, Hayer-Hartl M, Hartl FU (2013). Molecular chaperone functions in protein folding and proteostasis. Annu. Rev. Biochem..

[2] Balchin D, Hayer-Hartl M, Hartl FU (2016). In vivo aspects of protein folding and quality control. Science.

[3] Hartl FU, Bracher A, Hayer-Hartl M (2011). Molecular chaperones in protein folding and proteostasis. Nature.

[4] Preissler S, Deuerling E (2012). Ribosome-associated chaperones as key players in proteostasis. Trends Biochem. Sci..

[5] Hoffmann A, Bukau B, Kramer G (2010). Structure and function of the molecular chaperone Trigger Factor. Biochim. Biophys. Acta.

[6] Deuerling E, Schulze-Specking A, Tomoyasu T, Mogk A, Bukau B (1999). Trigger Factor and DnaK cooperate in folding of newly synthesized proteins. Nature.

[7] Oh E (2011). Selective ribosome profiling reveals the cotranslational chaperone action of Trigger Factor in vivo. Cell.

[8] Ferbitz L (2004). Trigger Factor in complex with the ribosome forms a molecular cradle for nascent proteins. Nature.

[9] Kramer G (2002). L23 protein functions as a chaperone docking site on the ribosome. Nature.

[10] Vogtherr M (2002). NMR solution structure and dynamics of the peptidyl-prolyl *cis-trans* isomerase domain of the Trigger Factor from *Mycoplasma genitalium* compared to FK506-binding protein. J. Mol. Biol..

[11] Yao Y, Bhabha G, Kroon G, Landes M, Dyson HJ (2008). Structure discrimination for the C-terminal domain of *Escherichia coli* Trigger Factor in solution. J. Biomol. NMR.

[12] Touw WG (2015). A series of PDB-related databanks for everyday needs. Nucleic Acids Res..

[13] Hafsa NE, Arndt D, Wishart DS (2015). CSI 3.0: a web server for identifying secondary and super-secondary structure in proteins using NMR chemical shifts. Nucleic Acids Res..

[14] Kaiser CM (2006). Real-time observation of Trigger Factor function on translating ribosomes. Nature.

[15] Patzelt H (2002). Three-state equilibrium of *Escherichia coli* Trigger Factor. Biol. Chem..

[16] Ludlam AV, Moore BA, Xu Z (2004). The crystal structure of ribosomal chaperone Trigger Factor from *Vibrio cholerae*. Proc. Natl Acad. Sci. USA.

[17] Martinez-Hackert E, Hendrickson WA (2009). Promiscuous substrate recognition in folding and assembly activities of the Trigger Factor chaperone. Cell.

[18] Kristensen O, Gajhede M (2003). Chaperone binding at the ribosomal exit tunnel. Structure.

[19] Martinez-Hackert E, Hendrickson WA (2007). Structures of and interactions between domains of Trigger Factor from *Thermotoga maritima*. Acta Crystallogr. D.

[20] Merz F (2008). Molecular mechanism and structure of Trigger Factor bound to the translating ribosome. EMBO J..

[21] Saio T, Guan X, Rossi P, Economou A, Kalodimos CG (2014). Structural basis for protein antiaggregation activity of the Trigger Factor chaperone. Science.

[22] Shi Y, Yu L, Kihara H, Zhou JM (2014). C-terminal 13-residue truncation induces compact trigger factor conformation and severely impairs its dimerization ability. Protein Pept. Lett..

[23] Zeng LL, Yu L, Li ZY, Perrett S, Zhou JM (2006). Effect of C-terminal truncation on the molecular chaperone function and dimerization of *Escherichia coli* Trigger Factor. Biochimie.

[24] Lakshmipathy SK (2007). Identification of nascent chain interaction sites on Trigger Factor. J. Biol. Chem..

[25] Hsu ST, Dobson CM (2009). ^1^H, ^15^N and ^13^C assignments of the dimeric ribosome binding domain of Trigger Factor from *Escherichia coli*. Biomol. NMR Assign..

[26] Rathore YS, Dhoke RR, Badmalia M, Sagar A, Ashish (2015). SAXS data based global shape analysis of Trigger Factor (TF) proteins from *E. coli*, *V. cholerae*, and *P. frigidicola*: resolving the debate on the nature of monomeric and dimeric forms. J. Phys. Chem. B.

[27] McConnell HM (1958). Reaction rates by nuclear magnetic resonance. J. Chem. Phys..

[28] Wüthrich K (1994). NMR assignments as a basis for structural characterization of denatured states of globular proteins. Curr. Opin. Struct. Biol..

[29] Rumpel S, Becker S, Zweckstetter M (2008). High-resolution structure determination of the CylR2 homodimer using paramagnetic relaxation enhancement and structure-based prediction of molecular alignment. J. Biomol. NMR.

[30] de Vries SJ, van Dijk M, Bonvin AM (2010). The HADDOCK web server for data-driven biomolecular docking. Nat. Protoc..

[31] van Zundert GC (2016). The Haddock2.2 web server: user-friendly integrative modeling of biomolecular complexes. J. Mol. Biol..

[32] Wassenaar TA (2012). WeNMR: structural biology on the grid. J. Grid Comput..

[33] Schwieters CD, Kuszewski JJ, Tjandra N, Clore GM (2003). The Xplor-NIH NMR molecular structure determination package. J. Magn. Reson..

[34] Parra RG (2016). Protein Frustratometer 2: a tool to localize energetic frustration in protein molecules, now with electrostatics. Nucleic Acids Res..

[35] Callon M, Burmann BM, Hiller S (2014). Structural mapping of a chaperone-substrate interaction surface. Angew. Chem. Int. Ed. Engl..

[36] Deeng J (2016). Dynamic behavior of Trigger Factor on the ribosome. J. Mol. Biol..

[37] Singhal K, Vreede J, Mashaghi A, Tans SJ, Bolhuis PG (2013). Hydrophobic collapse of Trigger Factor monomer in solution. PLoS. ONE.

[38] Thomas AS, Mao S, Elcock AH (2013). Flexibility of the bacterial chaperone Trigger Factor in microsecond-timescale molecular dynamics simulations. Biophys. J..

[39] He L, Sharpe T, Mazur A, Hiller S (2016). A molecular mechanism of chaperone-client recognition. Sci. Adv..

[40] Bond SR, Naus CC (2012). RF-Cloning.org: an online tool for the design of restriction-free cloning projects. Nucleic Acids Res..

[41] Benfield CT (2011). Mapping the IκB kinase β (IKKβ)-binding interface of the B14 protein, a vaccinia virus inhibitor of IKKβ-mediated activation of nuclear factor κB. J. Biol. Chem..

[42] Schuck P (2000). Size-distribution analysis of macromolecules by sedimentation velocity ultracentrifugation and lamm equation modeling. Biophys. J..

[43] Vistica J (2004). Sedimentation equilibrium analysis of protein interactions with global implicit mass conservation constraints and systematic noise decomposition. Anal. Biochem..

[44] Güntert P, Dötsch V, Wider G, Wüthrich K (1992). Processing of multi-dimensional NMR data with the new software PROSA. J. Biomol. NMR.

[45] Bartels C, Xia TH, Billeter M, Güntert P, Wüthrich K (1995). The program XEASY for computer-supported NMR spectral analysis of biological macromolecules. J. Biomol. NMR.

[46] Pervushin K, Riek R, Wider G, Wüthrich K (1997). Attenuated T_2_ relaxation by mutual cancellation of dipole-dipole coupling and chemical shift anisotropy indicates an avenue to NMR structures of very large biological macromolecules in solution. Proc. Natl Acad. Sci. USA.

[47] Salzmann M, Pervushin K, Wider G, Senn H, Wüthrich K (1998). TROSY in triple-resonance experiments: new perspectives for sequential NMR assignment of large proteins. Proc. Natl Acad. Sci. USA.

[48] Kjaergaard M, Poulsen FM (2011). Sequence correction of random coil chemical shifts: correlation between neighbor correction factors and changes in the Ramachandran distribution. J. Biomol. NMR.

[49] Xue Y (2009). Paramagnetic relaxation enhancements in unfolded proteins: theory and application to drkN SH3 domain. Protein Sci..

[50] Battiste JL, Wagner G (2000). Utilization of site-directed spin labeling and high-resolution heteronuclear nuclear magnetic resonance for global fold determination of large proteins with limited nuclear overhauser effect data. Biochemistry.

[51] Lee D, Hilty C, Wider G, Wüthrich K (2006). Effective rotational correlation times of proteins from NMR relaxation interference. J. Magn. Reson..

[52] Schumann FH (2007). Combined chemical shift changes and amino acid specific chemical shift mapping of protein-protein interactions. J. Biomol. NMR.

[53] Brunger AT (2007). Version 1.2 of the Crystallography and NMR system. Nat. Protoc..

[54] Brunger AT (1998). Crystallography & NMR system: a new software suite for macromolecular structure determination. Acta Crystallogr. D.

