# Supplementary material for: The dynamic dimer structure of the chaperone Trigger Factor
Source: Nat Commun. 2017 Dec 8;8:1992. doi: 10.1038/s41467-017-02196-7 (PMC5722895; doi:10.1038/s41467-017-02196-7)
Supplement: Supplementary file 1 — Supplementary Information [file 41467_2017_2196_MOESM1_ESM.pdf]

## Supplementary Figures

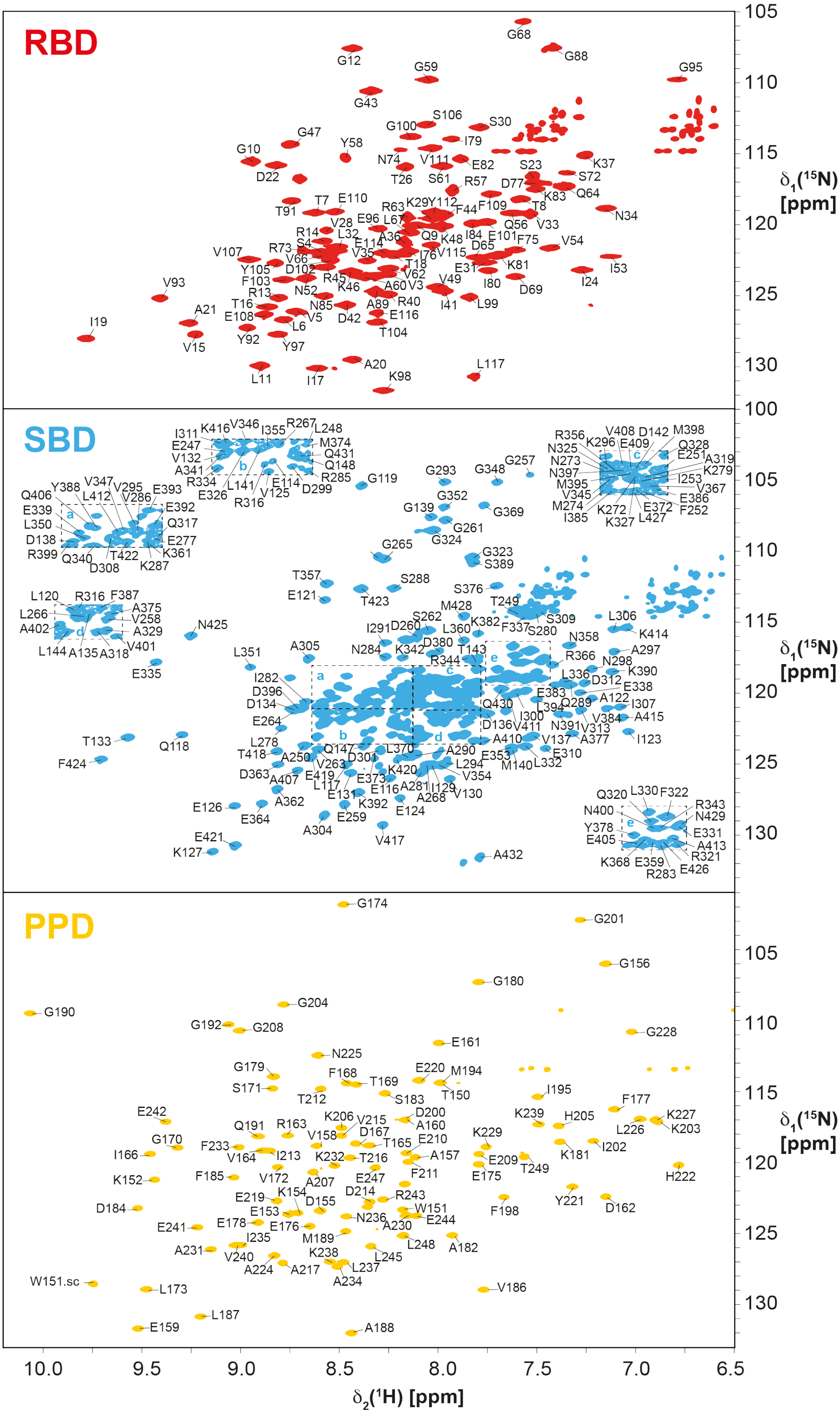


**Supplementary Figure 1 | Sequence-specific resonance assignment of TF domain constructs.** 2D [$^{15}$N,$^{1}$H]-TROSY spectrum of 100 µM [*U*-$^{15}$N] RBD, 250 µM [*U*-$^{2}$H,$^{15}$N] SBD, and 100 µM [*U*-$^{15}$N] PPD in sample buffer (20 mM K-phosphate pH 6.5, 100 mM KCl, 0.5 mM EDTA) at 25 °C and 700 MHz. The sequence-specific resonance assignments were obtained from 3D triple resonance experiments, and confirmed by comparison with published data (BMRB 15813 [1] and BMRB 19835–19837 [2]).

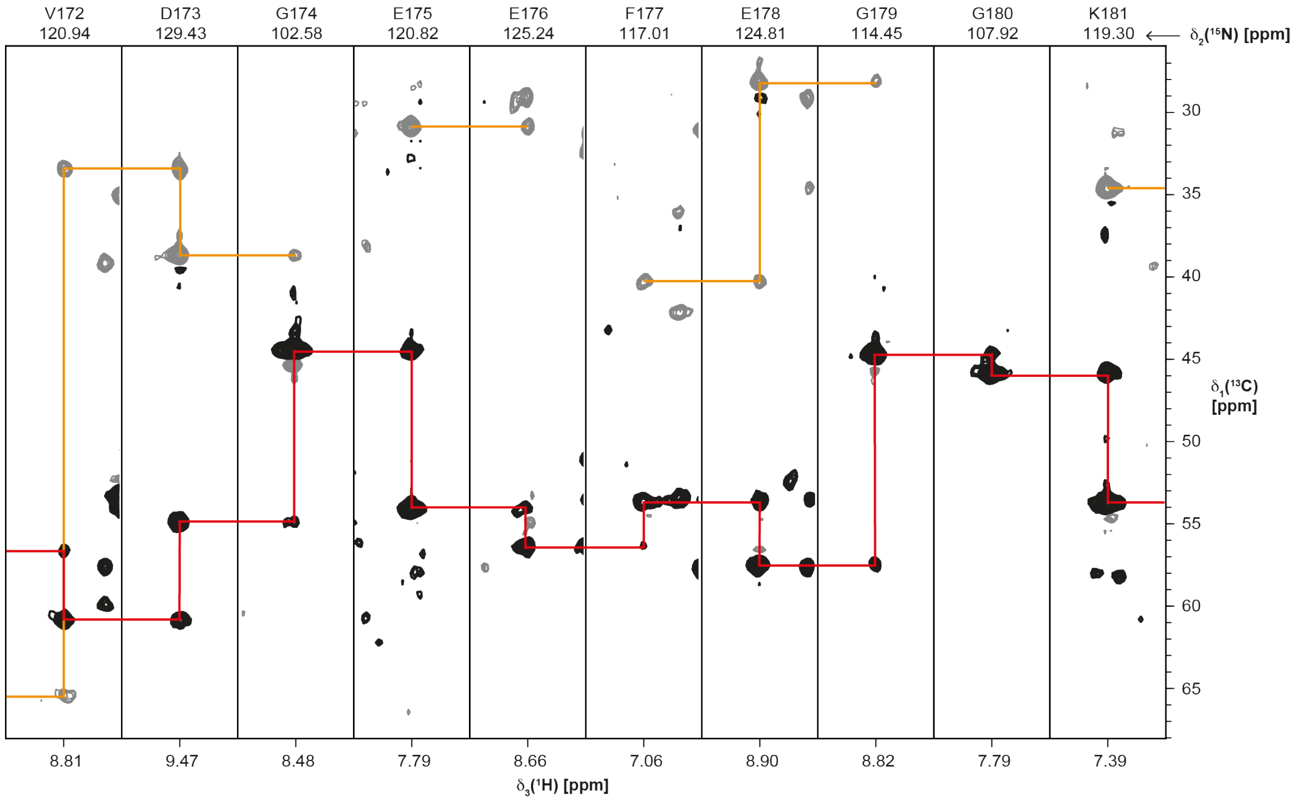


**Supplementary Figure 2 | Backbone NMR assignment of SBD–PPD.** Strips for residues 172–181 from a 3D TROSY-HNCACB experiment for [$U$-$^{2}$H,$^{15}$N,$^{13}$C] SBD–PPD acquired in sample buffer (20 mM K-phosphate pH 6.5, 100 mM KCl, 0.5 mM EDTA) at 25 °C and 700 MHz. The red and orange lines indicate the sequential connection between the strips for $^{13}C\alpha$ and $^{13}C\beta$ nuclei, respectively.

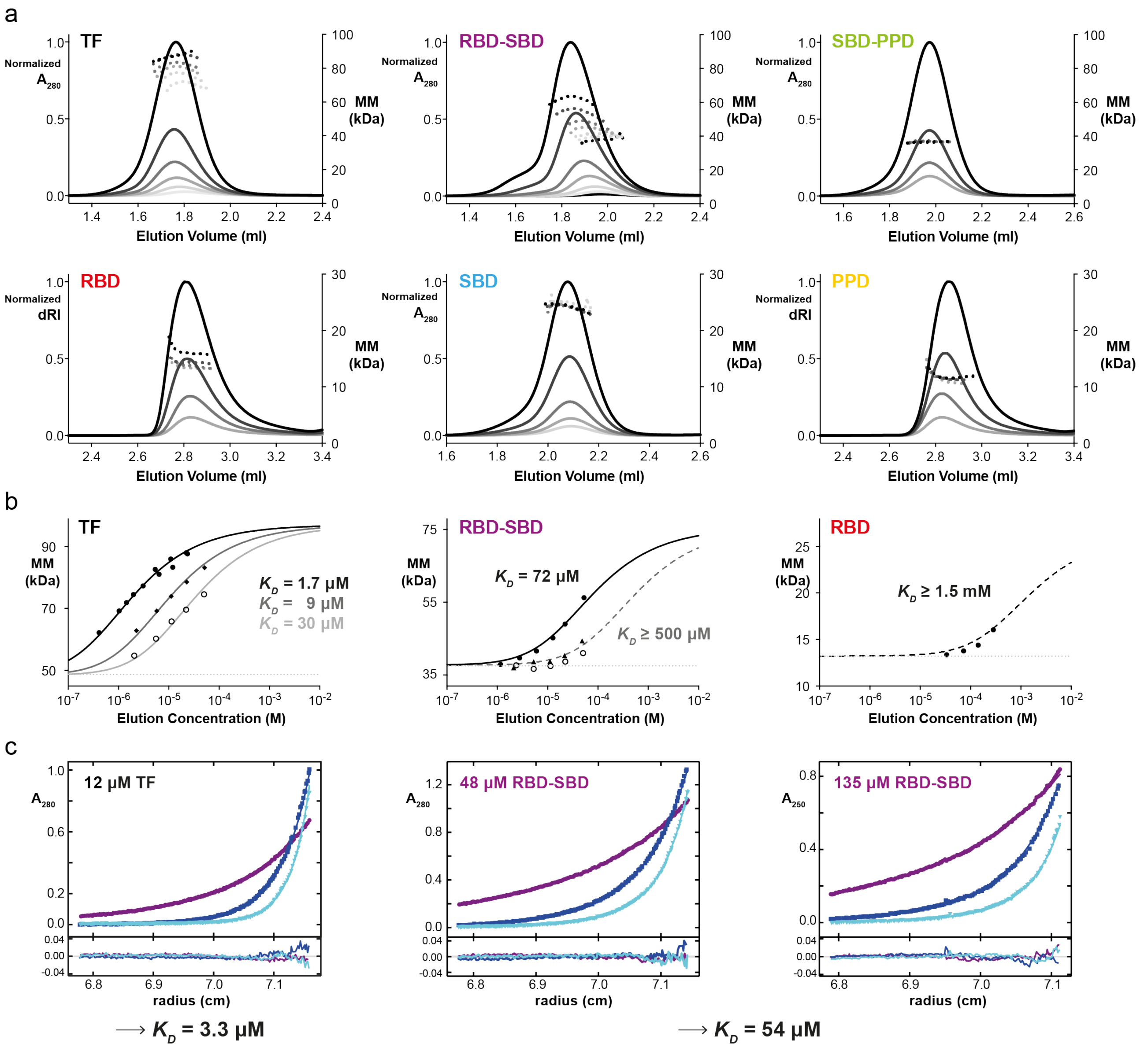


**Supplementary Figure 3 | Biophysical characterization of TF domain construct dimerizations.** Measurements were performed in sample buffer at 6 °C. **(a)** SEC elution profiles plotted as normalized absorbance ($A_{280}$) or normalized differential refractive index (dRI) (solid lines, left axis) and MALS apparent molecular mass (dotted lines, right axis) as a function of protein concentrations of the indicated domain constructs. **(b)** Nonlinear regression fit of the averaged molar mass as a function of the elution concentration to a monomer-dimer equilibrium [3]. For TF and RBD–SBD fittings are plotted for three different salt concentrations: 100 mM KCl (closed circles and black line), 250 mM KCl (closed diamonds and dark gray line) and 500 mM KCl (open circles and light gray line). **(c)** Analytical ultracentrifugation (AUC) profiles for 12 µM TF as well as 48 µM and 135 µM RBD–SBD. Data are shown for three centrifugation speeds (10, 16, and 20 krpm, in purple, blue, and cyan, respectively). For each construct, all data were globally fitted to a single monomer-dimer equilibrium. Residuals are shown below.

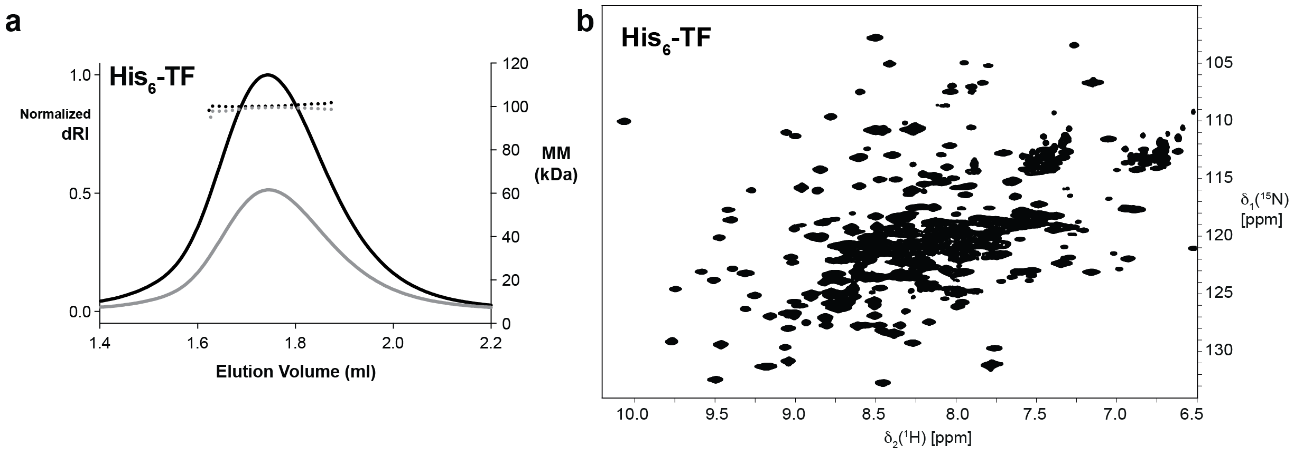


**Supplementary Figure 4 | Characterization of His$_6$-TF. (a)** SEC elution profile plotted as normalized differential refractive index (dRI) and MALS apparent molecular mass (dotted lines, right axis) as a function of protein concentrations. Elution concentration is 20 µM and 10 µM for black and gray lines, respectively, showing that the dimer dissociation constant $K_D$ must be below 100 nM. **(b)** 2D [$^{15}$N,$^{1}$H]-TROSY spectrum of 300 µM [$U$-$^{2}$H,$^{15}$N] His$_6$-TF in sample buffer (20 mM K-phosphate pH 6.5, 100 mM KCl, 0.5 mM EDTA) at 25 °C and 700 MHz.

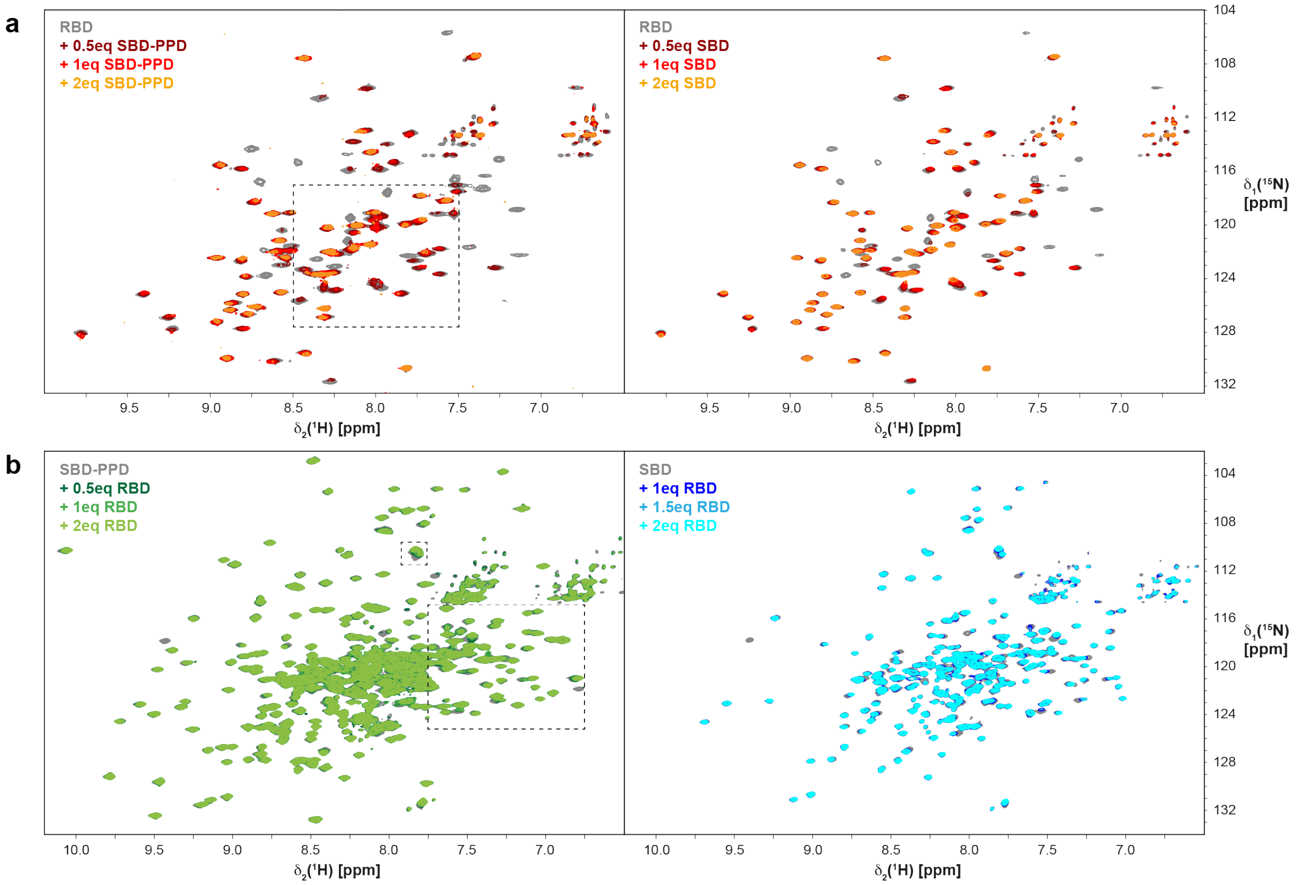


**Supplementary Figure 5 | NMR titrations of monomeric TF domains. (a)** Titration of unlabeled SBD–PPD to 100 µM [$U$-$^{15}$N] RBD (left) and of unlabeled SBD to 100 µM [$U$-$^{15}$N] RBD (right). **(b)** Titration of unlabeled RBD to 250 µM [$U$-$^{2}$H,$^{15}$N] SBD–PPD and of unlabeled RBD to 100 µM [$U$-$^{2}$H,$^{15}$N] SBD. The dashed rectangles correspond to the zoomed regions in Figure 3. Spectra were acquired in sample buffer (20 mM K-phosphate pH 6.5, 100 mM KCl, 0.5 mM EDTA) at 25 °C and 700 MHz.

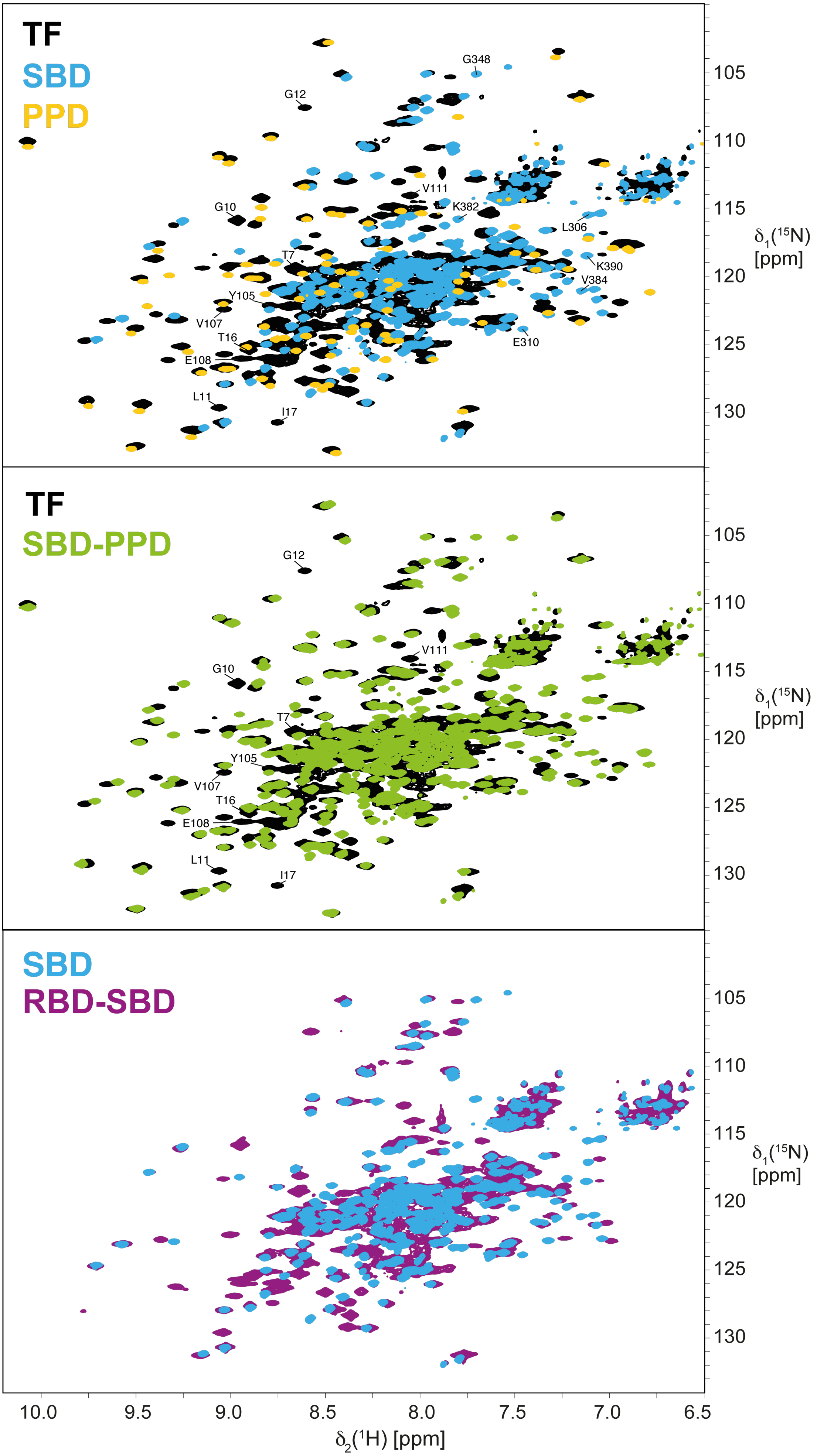
TF
SBD
PPD
G348
G12
V111
K382
G10
L306
K390
V384
T7
Y105
V107
T16
E108
E310
L11
I17
TF
SBD-PPD
SBD
RBD-SBD
105
110
115
120
125
130
δ1(15N) [ppm]
10.0
9.5
9.0
8.5
8.0
7.5
7.0
6.5
δ2(1H) [ppm]

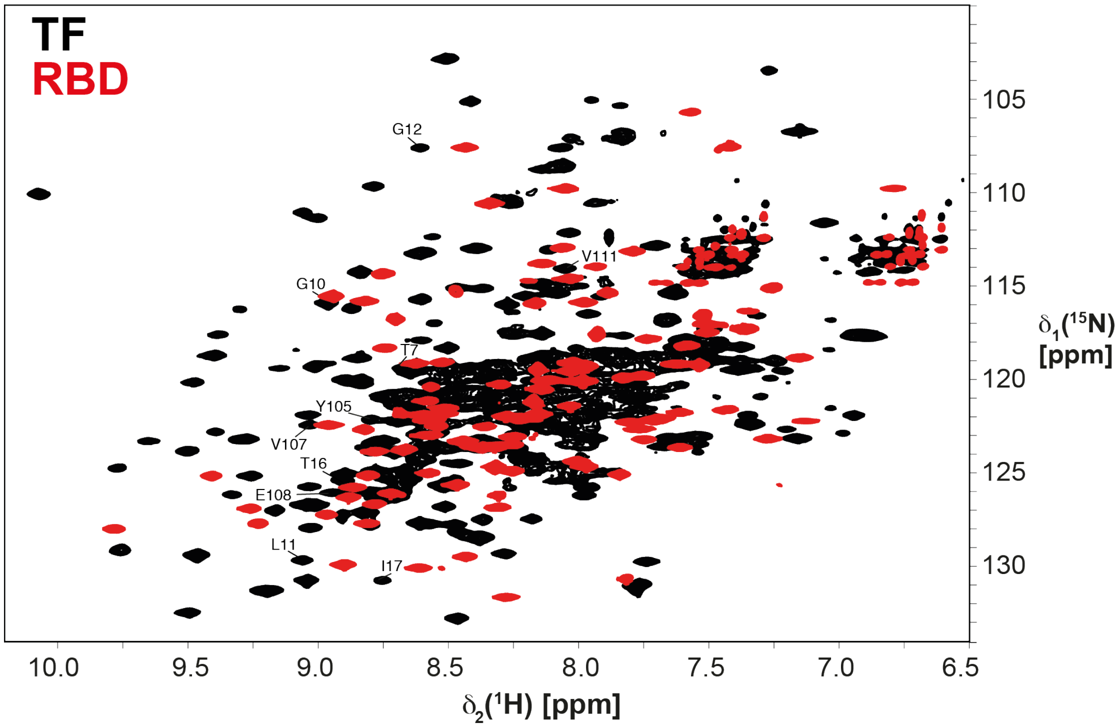


**Supplementary Figure 6 | NMR resonances of the RBD are line-broadened in full-length TF and RBD–SBD.** Overlays of 2D [$^{15}$N,$^{1}$H]-TROSY spectra of 500 µM [*U*-$^{2}$H,$^{15}$N] TF (black), [*U*-$^{2}$H,$^{15}$N] SBD (blue), [*U*-$^{15}$N] PPD (yellow), [*U*-$^{2}$H,$^{15}$N] SBD–PPD (purple), [*U*-$^{2}$H,$^{15}$N] RBD (red), and 250 µM [*U*-$^{2}$H,$^{15}$N] SBD–PPD (green). The spectra were acquired in sample buffer (20 mM K-phosphate pH 6.5, 100 mM KCl, 0.5 mM EDTA) at 25 °C and 700 MHz. Only 16 out of 108 expected RBD resonances are observed in full-length TF. Ten of these 16 resonances could be unambiguously assigned by comparison with the spectrum of the isolated RBD, together with the 3D TROSY HNCACB for [*U*-$^{2}$H,$^{15}$N,$^{13}$C]-TF. Several resonances of the SBD are line-broadened in TF full length, but not in the isolated SBD. The assignment of five of these resonances are indicated in the overlay of SBD / TF.

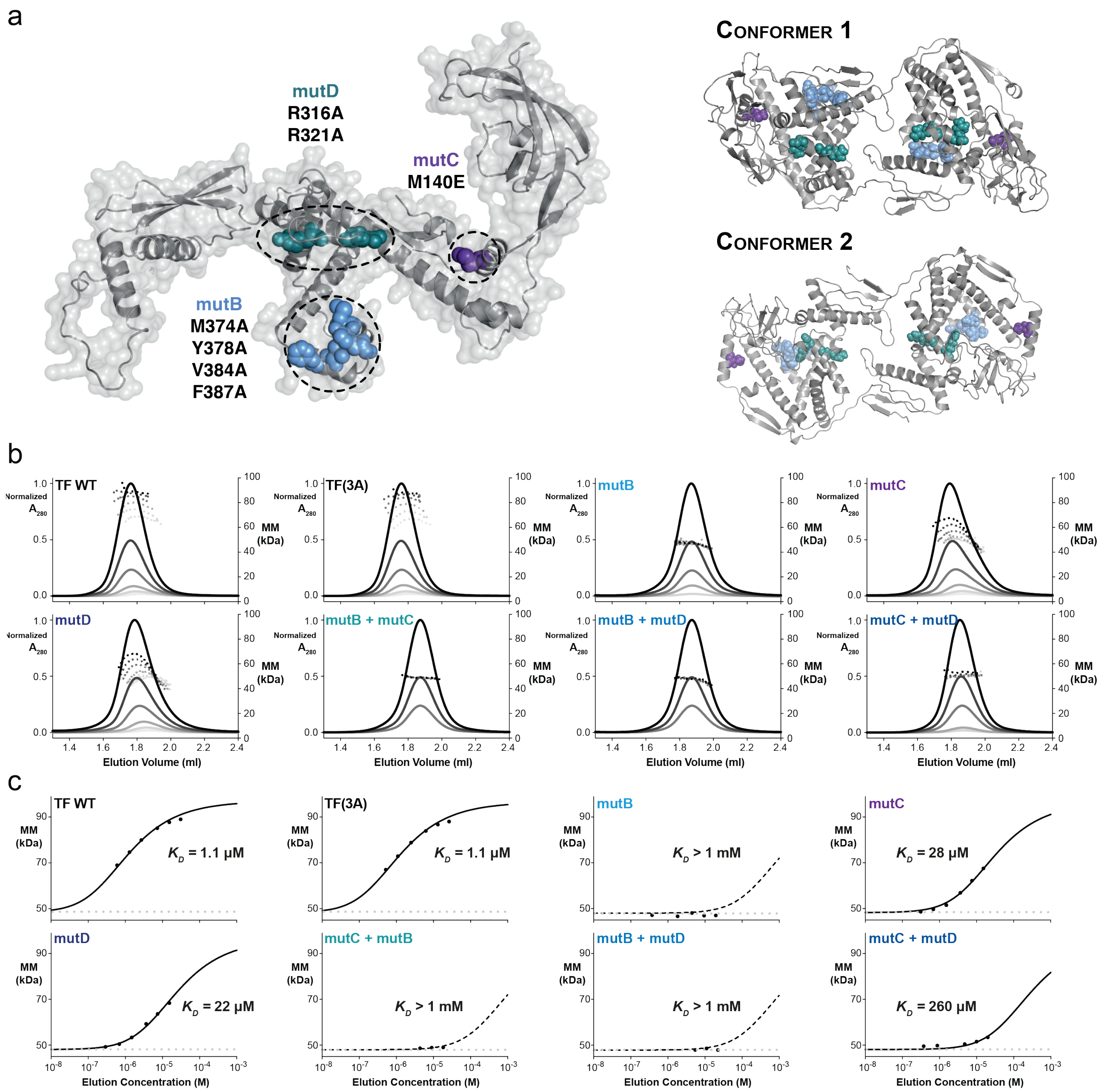


**Supplementary Figure 7 | Validation of the TF dimer structure by mutagenesis.** (**a**) Location of the mutation sets mutB, mutC, and mutD on the TF crystal structure (PDB 1W26) and conformers 1 and 2 of the TF dimer. (**b**) Biophysical characterization of TF mutants. Measurements were performed in sample buffer at 26°C. SEC elution profiles plotted as normalized absorbance ($A_{280}$) (solid lines, left axis) and MALS apparent molecular mass (dotted lines, right axis) as a function of protein concentrations of the indicated mutants. (**c**) Nonlinear regression fit of the averaged molar mass as a function of the elution concentration to a monomer-dimer equilibrium.

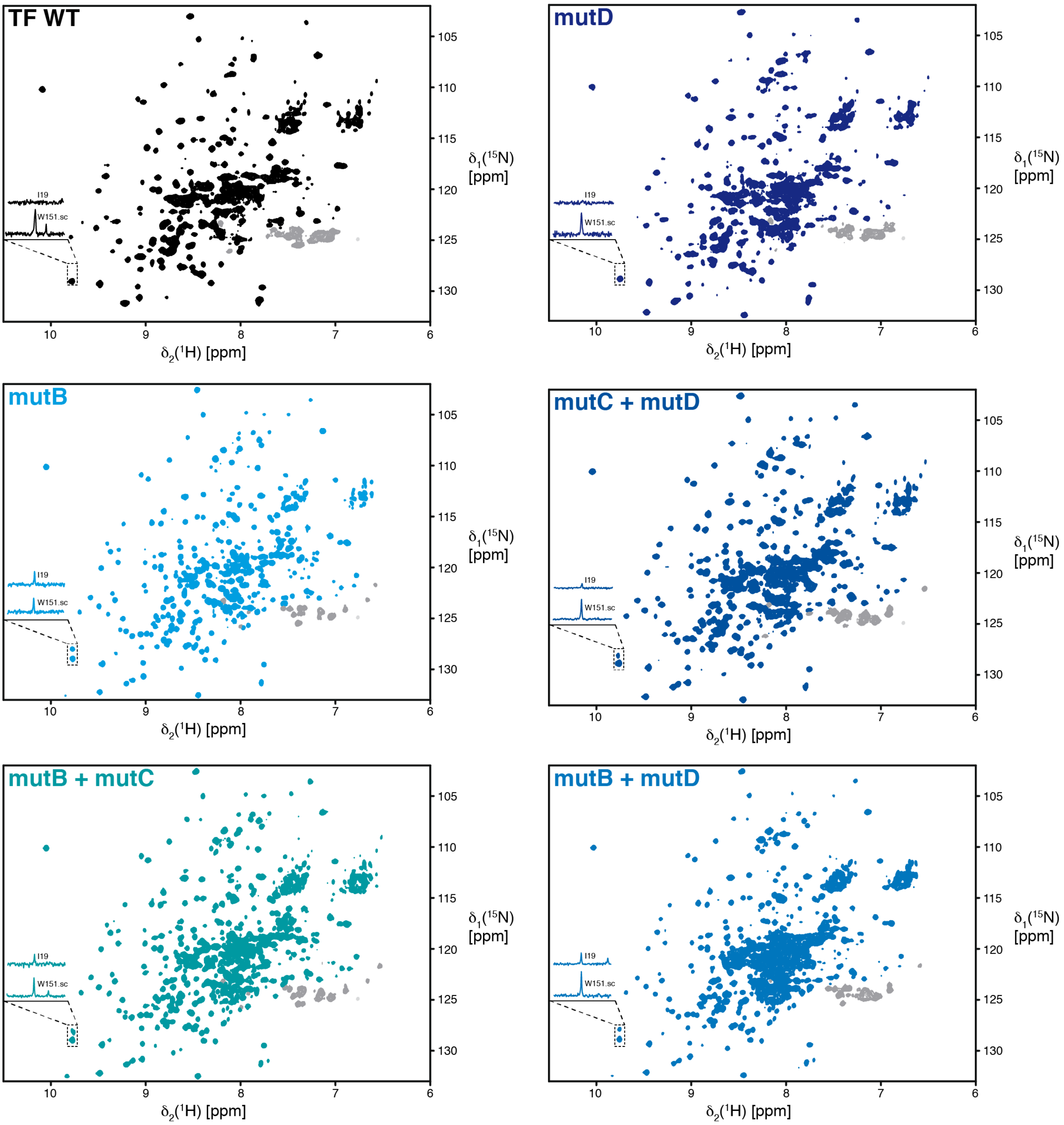


**Supplementary Figure 8 | Mutations at the dimer interface lower TF dimerization $K_D$.** 2D [$^{15}N$,$^{1}H$]-TROSY spectrum of 200 µM [*U*-$^{2}H$,$^{15}N$] TF and mutants acquired in sample buffer (20 mM K-phosphate pH 6.5, 100 mM KCl, 0.5 mM EDTA) at 25 °C and 700 MHz. In each spectrum a 1D-trace corresponding to W151 side-chain and to I19 is shown. The mutations lower the affinity between the protomers and peaks corresponding to residues that belong to the RBD become visible in the spectra.

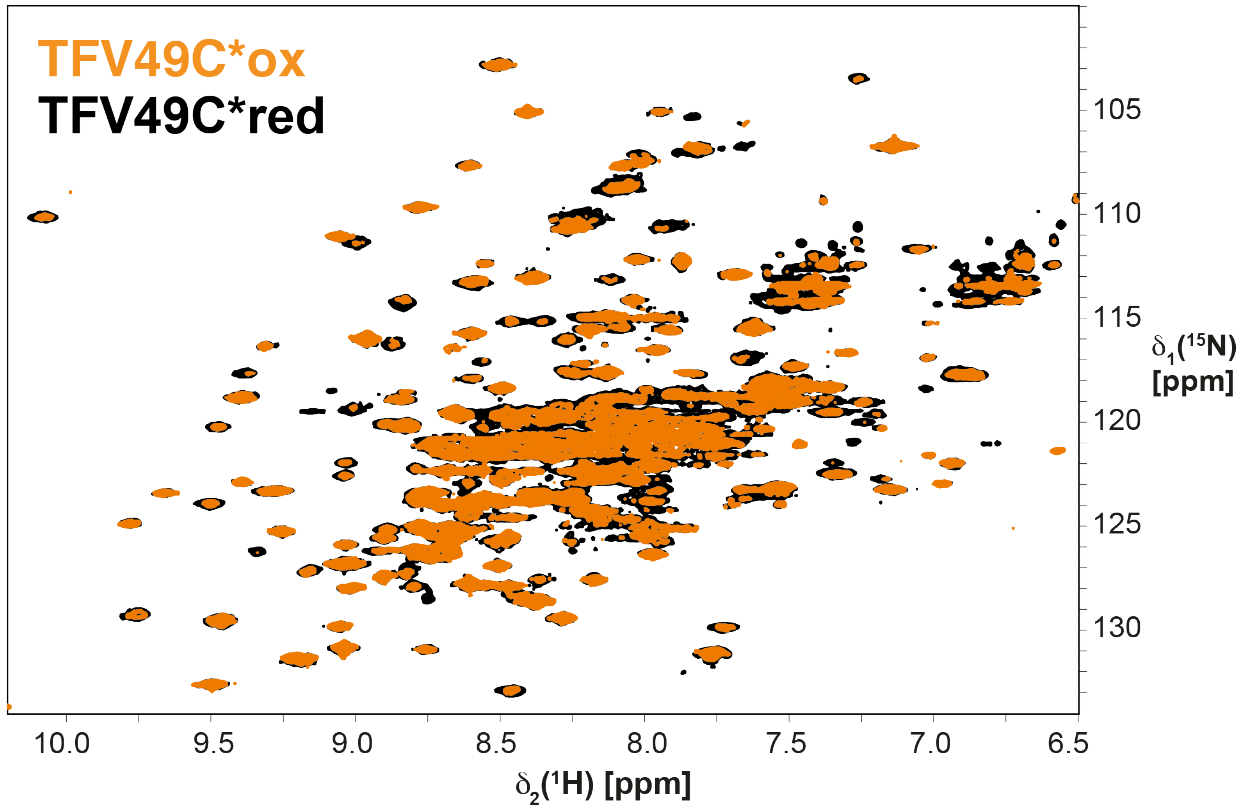


**Supplementary Figure 9 | Measurement of paramagnetic relaxation enhancement.** Overlay of 2D [$^{15}N$,$^{1}H$]-TROSY spectra of 300 µM [*U*-$^{2}H$,$^{15}N$] TF(V49C), spin labeled with MTSL in the paramagnetic oxidized state (orange) and the diamagnetic reduced state (black). Spectra were recorded in sample buffer (20 mM K-phosphate pH 6.5, 100 mM KCl, 0.5 mM EDTA) at 25 °C and 700 MHz.

**Supplementary Tables**

**Supplementary Table 1 | Interaction matrix of TF domain constructs[a].**

| | RBD | SBD | PPD | SBD–PPD | RBD–SBD | TF |
|---|---|---|---|---|---|---|
| **RBD** | – | + | – | + | n.d. | n.d. |
| **SBD** | + | – | – | – | n.d. | n.d. |
| **PPD** | – | – | – | – | n.d. | n.d. |
| **SBD–PPD** | + | – | – | – | n.d. | n.d. |
| **RBD–SBD** | n.d. | n.d. | n.d. | n.d. | + | n.d. |
| **TF** | n.d. | n.d. | n.d. | n.d. | n.d. | + |

[a]Diagonal entries were determined by SEC-MALS and AUC (Table 1) and off-diagonal entries by NMR spectroscopy. (+) = detectable interaction; (-) = no interaction detected; n.d. = not determined

**Supplementary Table 2 | Input parameters for HADDOCK docking and output parameters for the two structural clusters with highest ranks.**

| **HADDOCK input parameters** | | |
|---|---|---|
| *Number of active residues*[a] | | |
| Molecule A[b] | 38 | |
| Molecule B[c] | 64 | |
| *Symmetry restraints* | 432 | |
| **HADDOCK output** | | |
| | *Cluster 1* | *Cluster 2* |
| HADDOCK score | -153.7 ± 12.3 | -118.2 ± 16.4 |
| Cluster size | 9 | 22 |
| Average pairwise backbone RMSD within the cluster | 0.94 ± 0.34 | 0.47 ± 0.12 |
| Backbone RMSD between the lowest-energy structures | 15.8 | |

[a] residues for which significant line-broadening or significant chemical shift perturbation was observed upon interaction

[b] active residues on RBD

[c] active residues on SBD

**Supplementary Table 3 | Input data for structure refinement with XPLOR-NIH.**

| **Constraint type** | **Amount** | |
|---|---|---|
| *EFN restraint* | 3116/monomer | |
| *PRE restraint* | *Intra- or intermolecular A-A, B-B or A-B, B-A (medium distance)* | *Intra- and intermolecular A-A, B-B, A-B, B-A (long distance)* |
| S30C | 6 | 95 |
| V49C | 63 | 32 |
| S61C | 51 | 32 |
| S72C | 10 | 75 |
| A223C | 27 | 45 |
| E326C | 14 | 71 |
| **Total** | **171** | **350** |

**Supplementary Table 4 | XPLOR-NIH structural statistics.**

| **XPLOR-NIH output** | | |
|---|---|---|
| *Structure statistics* [a] | *Conformer 1* | *Conformer 2* |
| Intramolecular restraints | 42 | 44 |
| Intermolecular restraints | 129 | 127 |
| | | |
| *Violations long-distance PRE restraints* [a] | | |
| Number of violations (>0.5 Å) | | |
| Intramolecular | 1 | 0 |
| Intermolecular | 4 | 1 |
| Average violations (Å) | | |
| Intramolecular | 2.64 | - |
| Intermolecular | 1.42 ± 0.75 | 0.57 |
| Max. PRE distance constraint violation (Å) | | |
| Intramolecular | 2.64 | - |
| Intermolecular | 2.47 | 0.57 |
| *Violations medium-distance PRE restraints* [a] | | |
| Number of violations (>0.5 Å) | | |
| Intramolecular | 14 | 16 |
| Intermolecular | 33 | 29 |
| Average violations (Å) | | |
| Intramolecular | 1.52 ± 0.97 | 1.80 ± 1.03 |
| Intermolecular | 1.90 ± 1.25 | 1.63 ± 1.01 |
| Max. PRE distance constraint violation (Å) | | |
| Intramolecular | 3.21 | 4.43 |
| Intermolecular | 5.17 | 4.97 |
| | | |
| Deviations from idealized geometry [a] | | |
| Bond lengths (Å) | 0.005 | 0.005 |
| Bond angles (°) | 0.539 | 0.572 |
| Impropers (°) | 0.841 | 0.965 |
| | | |
| Ramachandran analysis [b] | | |
| Most favored regions (%) | 90.8 | 86.6 |
| Additional allowed regions (%) | 7.6 | 9.9 |
| Generously allowed regions (%) | 1.0 | 1.6 |
| Disallowed regions (%) | 0.5 | 1.9 |
| | | |
| Average pairwise rmsd (Å) [c] | | |
| Heavy atoms | 2.13 ± 0.73 | 1.74 ± 0.79 |
| Backbone atoms | 2.04 ± 0.74 | 1.66 ± 0.79 |

[a] Calculated for the lowest energy structures
[b] Calculated with PROCHECK-NMR for the ensemble of the 10 lowest energy structures
[c] Pairwise rmsd calculated with PyMOL between the 10 lowest energy structures

**Supplementary Table 5 | Dimer dissociation constants of TF mutants.**

| **Mutant sets**[a] | $K_D$ **(μM)** |
|---|---|
| – | $1.1 \pm 0.2$ |
| mutB | > 1000 |
| mutC | $28 \pm 4$ |
| mutD | $22 \pm 3$ |
| mutD + mutC | $260 \pm 130$ |
| mutB + mutC | > 1000 |
| mutB + mutD | > 1000 |

[a] introduced on the TF(3A) background

**Supplementary Table 6 | Primer sequences used for cloning.**

| Primer | Sequence |
|---|---|
| **TF_fw** | GGAATTCCATATGCAAGTTTCAGTTGAAACC |
| **TF_rev** | ACACGCGGCCGCTTACGCCTCTGGTTCATCAGC |
| **TF_117_Stop_fw** | CCGGAAGTTGAACTGTAGGGTCTGGAAGCGA |
| **TF_117_Stop_rev** | TCGCTTCCAGACCCTACAGTTCAACTTCCGG |
| **TF_RF_114fw** | GGTGCCGCGCGGCAGCCATATGGAAGTTGAACTGCAGGGTCT |
| **TF_RF_432rev** | GGTGCTCGAGTGCGGCCGCTTACGCCTGCTGGTTCATCAG |
| **TF_RF_fw** | GTGCCGCGCGGCAGCCATATGCAAGTTTCAGTTGAAACCAC |
| **TF_RF_149rev** | GGTGGTGGTGGTGGTGCTCGAGCTGCTGTTTACGCAGAGTATCC |
| **TF_RF_149/249_fw** | CGGCATGCTGGATACTCTGCGTAAACAGCAGGAACTGACTGCAGAATTCATCAAA |
| **TF_RF_149fw** | GGTGCCGCGCGGCAGCCATATGGCGACCTGGAAAGAAAAAGAC |
| **TF_RF_249rev** | GGTGCTCGAGTGCGGCCGCTTAAGTCAGTTCCGGCAGTTC |
| **Thr2TEV_fw** | GCCATCATCATCATCATCACAGCGAGAACCTATATTTCCAGGGCAGCCATATG |
| **Thr2TEV_rev** | CATATGGCTGCCCTGGAAATATAGGTTCTCGCTGTGATGATGATGATGATGGC |
| **TF_S30C_fw** | GACCAGCTCGCATTTAACAGCGGTCTCGATGCT |
| **TF_S30C_rev** | GACCAGCTCGCATTTAACAGCGGTCTCGATGCT |
| **TF_V49C_fw** | TTGACGGCTTCCGCAAAGGCAAATGCCCAATGAATATCGTTGCTC |
| **TF_V49C_rev** | GAGCAACGATATTCATTGGGCATTTGCCTTTGCGGAAGCCGTCAA |
| **TF_S61C_fw** | GCGTTATGGCGCGTGTGTACGCCAGG |
| **TF_S61C_rev** | CCTGGCGTACACACGCGCCATAACGC |
| **TF_S72C_fw** | TTCTGGGTGACCTGATGTGCCGTAACTTCATTGAC |
| **TF_S72C_rev** | GTCAATGAAGTTACGGCACATCAGGTCACCCAGAA |
| **TF_A223C_fw** | ACCTTCCCGGAAGAATACCACTGCGAAAACCTGAAAGGTAAAGCA |
| **TF_A223C_rev** | TGCTTTACCTTTCAGGTTTTCGCAGTGGTATTCTTCCGGGAAGGT |
| **TF_E326C_fw** | CACAGCGTTTCGGTGGCAACTGCAAACAAGCTCTGGAACTGCC |
| **TF_E326C_rev** | GGCAGTTCCAGAGCTTGTTTGCAGTTGCCACCGAAACGCTGTG |
| **TF_FRK44-46_AAA_fw** | GTTGCGAAAAAAGTACGTATTGACGGCGCCGCCGCAGGCAAAGTGCCAATGAATATCGTTGC |
| **TF_FRK44-46_AAA_rev** | GCAACGATATTCATTGGCACTTTGCCTGCGGCGGCGCCGTCAATACGTACTTTTTTCGCAAC |
| **TF_R321A_fw** | GCCAGGCTGCACAGGCTTTCGGTGGCAACG |
| **TF_R321A_rev** | CGTTGCCACCGAAAGCCTGTGCAGCCTGGC |
| **TF_R316A_fw** | GCGAAATCGACGTTCTGCGTGCCCAGGCTGCAC |
| **TF_R316A_rev** | GTGCAGCCTGGGCACGCAGAACGTCGATTTCGC |
| **TF_M140E_fw** | GCTGACGTTGACGGCGAGCTGGATACTCTGCG |
| **TF_M140E_rev** | CGCAGAGTATCCAGCTCGCCGTCAACGTCAGC |
| **TF_V384A_F387A_fw** | GCGTACGAAGATCCGAAAGAAGCTATCGAGGCCTACAGCAAAAACAAAGAACTG |
| **TF_V384A_F387A_rev** | CAGTTCTTTGTTTTTGCTGTAGGCCTCGATAGCTTCTTTCGGATCTTCGTACGC |
| **TF_M374A_Y378A_fw** | AAGGCCTGATCGAAGAGGCGGCTTCTGCGGCCGAAGATCCGAAAGAAG |
| **TF_M374A_Y378A_rev** | CTTCTTTCGGATCTTCGGCCGCAGAAGCCGCCTCTTCGATCAGGCCTT |

## Supplementary Notes

### Supplementary Note 1

**Kinetic equations for the determination of the lifetime of the TF dimer**

The monomer-dimer equilibrium for the two differently labeled forms of TF is described by the following chemical equilibrium reactions

$$2\mathrm{N} \rightleftarrows \mathrm{N}_2 \quad (1)$$

$$\mathrm{N} + \mathrm{S} \rightleftarrows \mathrm{NS} \quad (2)$$

$$2\mathrm{S} \rightleftarrows \mathrm{S}_2 \quad (3)$$

where N and S are the $^{15}$N-labeled and spin-labeled forms of monomeric TF, respectively. The three reactions 1–3 have the same $k_{\mathrm{off}}$, $k_{\mathrm{on}}$ and $K_{\mathrm{d}}$ values. The experimental conditions were chosen to have equal amounts of the two labeled forms:

$$[\mathrm{N}]_{\mathrm{total}} = [\mathrm{S}]_{\mathrm{total}} \quad (4)$$

At $t$ = 0, equal samples of pure N and pure S are mixed and the equations hold:

$$[\mathrm{N}_2]_0 = [\mathrm{S}_2]_0 = c \ \text{ and } \ [\mathrm{NS}]_0 = 0 \quad (5)$$

The symmetry argument ($K_{\mathrm{d}}$ is the same for $\mathrm{N}_2$, $\mathrm{S}_2$, and NS) yields that at all times

$$[\mathrm{N}_2] = [\mathrm{S}_2] \quad (6)$$

Furthermore, symmetry considerations yield that at all times

$$[\mathrm{N}] = [\mathrm{S}] = \mathrm{const} \quad (7)$$

and

$$[\mathrm{N}_2] + [\mathrm{NS}] + [\mathrm{S}_2] = 2c \quad \text{, rewritten as} \quad [\mathrm{NS}] = 2c - 2[\mathrm{N}_2] \quad (8)$$

After mixing of the samples, the system evolves from

$$[\mathrm{N}_2]_0 = [\mathrm{S}_2]_0 = c \ \text{ and } \ [\mathrm{NS}]_0 = 0 \quad \text{at } t = 0 \quad (5)$$

to

$$[\mathrm{N}_2]_{\mathrm{eq}} = [\mathrm{S}_2]_{\mathrm{eq}} = [\mathrm{NS}]_{\mathrm{eq}} = \tfrac{2}{3}c \quad \text{at } t = \infty \quad (9)$$

The rate equations for the evolution of the system from $t$ = 0 to $t = \infty$ are given by

$$\frac{\mathrm{d[N_2]}}{\mathrm{d}t} = -k_{\mathrm{off}}[\mathrm{N_2}] + k_{\mathrm{on}}[\mathrm{N}]^2 \qquad (10)$$

$$\frac{\mathrm{d[NS]}}{\mathrm{d}t} = -k_{\mathrm{off}}[\mathrm{NS}] + k_{\mathrm{on}}[\mathrm{N}][\mathrm{S}] \qquad (11)$$

combining eqs. 10 and 11 yields

$$\frac{\mathrm{d[N_2]}}{\mathrm{d}t} - \frac{\mathrm{d[NS]}}{\mathrm{d}t} = -k_{\mathrm{off}}[\mathrm{N_2}] + k_{\mathrm{off}}[\mathrm{NS}] + k_{\mathrm{on}}[\mathrm{N}]^2 - k_{\mathrm{on}}[\mathrm{N}][\mathrm{S}]$$

$$\frac{\mathrm{d[N_2]}}{\mathrm{d}t} - \frac{\mathrm{d}(2c-2[\mathrm{N_2}])}{\mathrm{d}t} = -k_{\mathrm{off}}[\mathrm{N_2}] + k_{\mathrm{off}}[\mathrm{NS}] + k_{\mathrm{on}}[\mathrm{N}]^2 - k_{\mathrm{on}}[\mathrm{N}][\mathrm{N}]$$

$$\frac{\mathrm{d[N_2]}}{\mathrm{d}t} + 2\frac{\mathrm{d[N_2]}}{\mathrm{d}t} = -k_{\mathrm{off}}[\mathrm{N_2}] + k_{\mathrm{off}}(2c - 2[\mathrm{N_2}])$$

$$\frac{3\mathrm{d[N_2]}}{\mathrm{d}t} = -k_{\mathrm{off}}(3[\mathrm{N_2}] - 2c)$$

$$\frac{3\mathrm{d[N_2]}}{(3[\mathrm{N_2}]-2c)} = -k_{\mathrm{off}}\mathrm{d}t$$

$$\frac{\mathrm{d[N_2]}}{[\mathrm{N_2}]-\frac{2}{3}c} = -k_{\mathrm{off}}\mathrm{d}t \qquad (12)$$

Integration yields

$$\int_c^{[\mathrm{N_2}](t)} \frac{\mathrm{d[N_2]}}{[\mathrm{N_2}]-\frac{2}{3}c} = -k_{\mathrm{off}}\int_0^t \mathrm{d}t$$

$$\ln\left([\mathrm{N_2}](t) - \frac{2}{3}c\right) - \ln\left(c - \frac{2}{3}c\right) = -k_{\mathrm{off}}t$$

$$\ln\left(\frac{[\mathrm{N_2}](t)-\frac{2}{3}c}{\frac{1}{3}c}\right) = -k_{\mathrm{off}}t$$

$$\frac{[\mathrm{N_2}](t)-\frac{2}{3}c}{\frac{1}{3}c} = \exp\left(-k_{\mathrm{off}}t\right) \qquad (13)$$

With the final result

$$[\mathrm{N_2}](t) = \frac{1}{3}c\exp(-k_{\mathrm{off}}t) + \frac{2}{3}c$$

$$[\mathrm{S_2}](t) = \frac{1}{3}c\exp(-k_{\mathrm{off}}t) + \frac{2}{3}c \qquad (14)$$

$$[\mathrm{NS}](t) = \frac{2}{3}c - \frac{2}{3}c\exp(-k_{\mathrm{off}}t)$$

Supplementary Figure 10 shows a numeric simulation of eqs. 14.

The observed NMR signal in the lifetime experiment is given by

$$I(t) = a_1[\mathrm{N_2}](t) + a_2[\mathrm{NS}](t) = (\frac{1}{3}a_1 - \frac{2}{3}a_2)\cdot c\exp(-k_{\mathrm{off}}t) + (a_1 + a_2)\frac{2}{3}c \qquad (15)$$

where $a_1$ and $a_2$ are the specific molar NMR intensities of the $N_2$ and NS species, respectively. Since $a_1$, $a_2$ and $c$ are constants, eq. 15 can be rewritten as

$$I(t) = (I_0 - I_\infty)\cdot\exp(-k_{\mathrm{off}}t) + I_\infty \qquad (16)$$

where the constants $I_0$ and $I_\infty$ are the signal intensities at $t = 0$ and $t = \infty$, respectively. The off-rate constant $k_{\mathrm{off}}$ can be obtained from non-linear fits of experimental data to eq. 16 or to the equivalent form

$$I_\Delta(t) = I(t) - I_\infty = (I_0 - I_\infty)\cdot\exp(-k_{\mathrm{off}}t) \qquad (17)$$

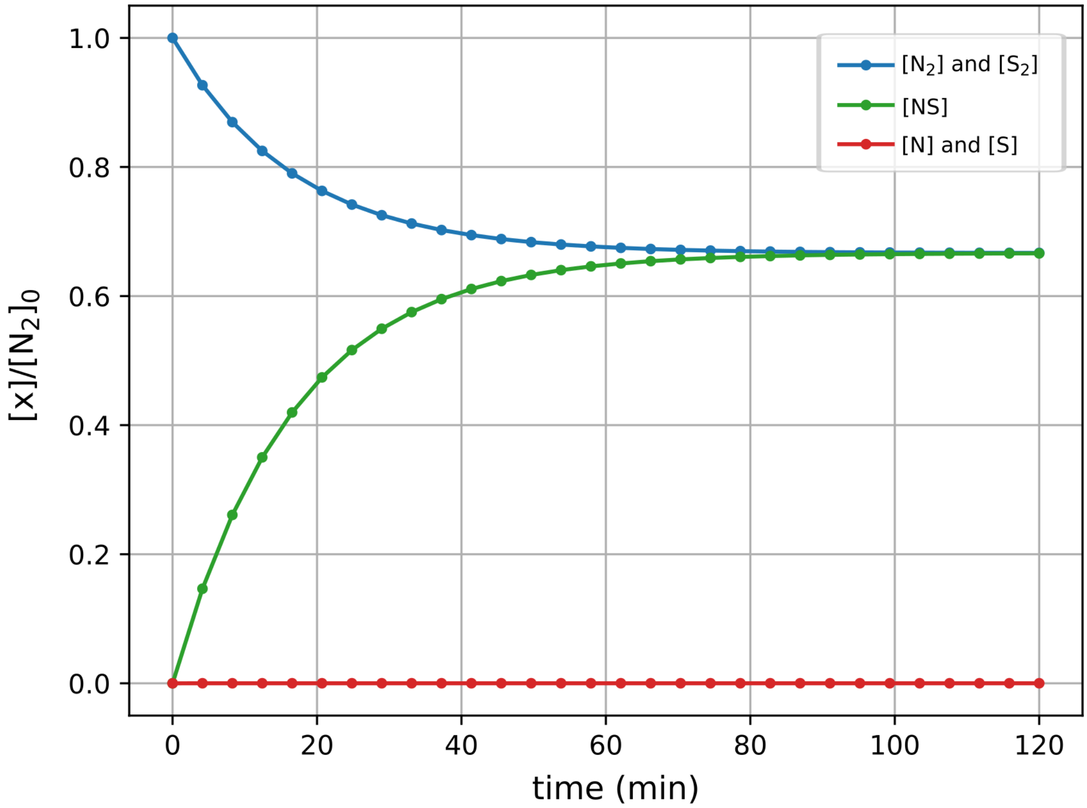


**Supplementary Figure 10 |** Numerical simulation integration of equations 11 and 12 with parameters $K_d$ = 2µM, $k_{off}$ = 0.001 $s^{-1}$, $[N]_{total}$ = 100 µM.

## Supplementary References


1. Hsu, S.T. & Dobson, C.M. $^{1}H$, $^{15}N$ and $^{13}C$ assignments of the dimeric ribosome binding domain of Trigger Factor from *Escherichia coli*. *Biomol. NMR Assign.* **3**, 17–20 (2009).
2. Saio, T., Guan, X., Rossi, P., Economou, A. & Kalodimos, C.G. Structural basis for protein antiaggregation activity of the Trigger Factor chaperone. *Science* **344**, 1250494 (2014).
3. Benfield, C.T. *et al.* Mapping the IκB kinase β (IKKβ)-binding interface of the B14 protein, a vaccinia virus inhibitor of IKKβ-mediated activation of nuclear factor κB. *J. Biol. Chem.* **286**, 20727–20735 (2011).
